# Activity Coefficients of HCl in Solutions Related to “Tris” Buffers in Artificial Seawater. II. HCl + NaCl + TrisHCl + H_2_O, and Tris Buffer + NaCl + H_2_O, to High Ionic Strength and from 5 to 40 °C

**DOI:** 10.1021/acs.jced.5c00369

**Published:** 2025-08-11

**Authors:** Igor Maksimov, Toshiaki Asakai, Yuya Hibino, Simon L. Clegg

**Affiliations:** † National Metrology Institute of Japan, 119882 National Institute of Advanced Industrial Science and Technology (AIST), 1-1-1 Umezono, Tsukuba, Ibaraki 305-8563, Japan; ‡ School of Environmental Sciences, 6106 University of East Anglia, Norwich NR4 7TJ, United Kingdom

## Abstract

The substance Tris (2-amino-2-hydroxymethyl-1,3-propanediol, CAS 77-86-1), and its protonated form TrisH^+^, are used in the preparation of ‘total’ pH buffers in artificial seawater media. The development of a chemical speciation model of the buffer solutions, using the Pitzer equations to calculate solute activity coefficients, is desirable in order to quantify the effects of composition change, convert the total pH to other scales, and address metrological requirements for traceability to the International System of Units. Here, in the second of a series of studies, we present Harned cell measurements of potentials and mean activity coefficients of HCl in solutions containing HCl, NaCl, and TrisHCl for ionic strengths from 1.0 to 5.5 mol kg^–1^ and from 5 to 40 °C. The results at 25 °C are consistent with those of the literature studies of the two end-member solutions (aqueous HCl + NaCl, and HCl + TrisHCl). We also present results of measurements of buffer solutions containing equimolal Tris and TrisHCl (hence TrisH^+^), and NaCl, at ionic strengths of 0.2, 1.0, and 4.0 mol kg^–1^ at the same temperatures. These are compared with literature data for Tris buffers in an artificial seawater medium. Aspects of the development of a Pitzer model for these solutions are discussed.

## Graphical abstract

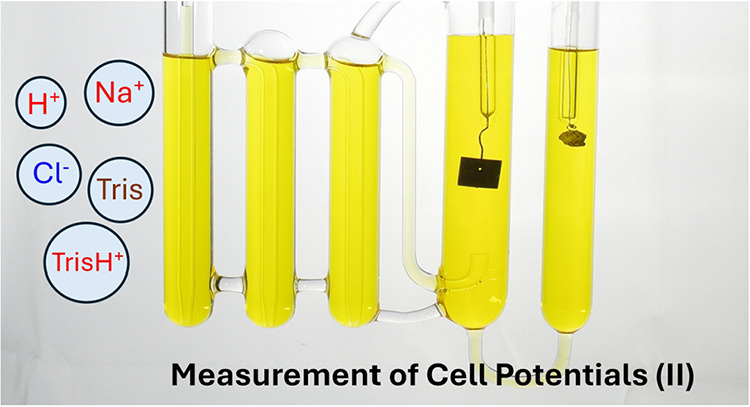

## 1 Introduction

The seawater total hydrogen ion pH scale was established from measurements of cell potentials of solutions of artificial seawater acidified with HCl, and others containing equimolal Tris and its conjugate acid TrisH^+^ as a pH buffer.^1^ (The substance Tris is 2-amino-2-hydroxymethyl-1,3-propanediol, and the acidic form TrisH^+^ has a p*K*_a_ that is close to the pH of seawater.) Artificial seawaters, and natural seawater, consist of about 90 mol % Na^+^ and Cl^–^ ions, plus smaller amounts of Mg^2+^, SO_4_^2–^, Ca^2+^, and K^+^. Other minor species present in natural seawater^2^ are generally omitted from artificial seawaters because their molalities are too low to influence the activity coefficients of other solutes. The development of a chemical speciation model of these buffer solutions, yielding molalities and activities of solute species for a range of salinities and temperatures, and hence total pH, has a number of potential benefits. These include the extension of the scale to a wider range of temperatures and salinities, conversion to other forms such as “free” pH^3^ and conventional pH,^4^ and improved metrological traceability.^5–7^

Clegg et al.^6^ have developed a draft model of Tris buffer in artificial seawater, using the Pitzer equations^8^ for the calculation of activity coefficients, and have tabulated the unknown Pitzer interaction parameters that new thermodynamic data are needed to quantify. As the first step toward achieving this goal, Maksimov et al.^7^ have determined mean activity coefficients of HCl in aqueous HCl–TrisHCl mixtures from measured potentials of Harned cells (which yield activity products of H^+^ and Cl^–^). This study is the second of a series which involves the National Metrology Institutes of Japan (hereinafter NMIJ), Germany, and the USA. Here we present measurements of electrochemical potentials of aqueous HCl–NaCl–TrisHCl mixtures, and Tris buffer in aqueous NaCl solutions, over a range of temperatures and ionic strengths as a further step toward developing a model of the pH buffer. The results are compared with available data for aqueous HCl–TrisHCl and HCl–NaCl solutions and for Tris buffer in artificial seawater.

## 2 Experimental Method

Activity products of H^+^ and Cl^–^ ions were determined from measurements of the potential difference of the following electrochemical cell:

$${\mathrm{Pt}}_{\left(s\right)},\;H_{2(g)}\left(1\;\mathrm{atm}\right)\left|H^{+},\;{\mathrm{Cl}}^{-}\;\mathrm{in aq}.\;\mathrm{soln}.\right|{\mathrm{Ag}}_{\left(s\right)}/{\mathrm{AgCl}}_{(s)}$$ A

where the solutions in this study contain either H^+^, Na^+^, TrisH^+^ and Cl^–^ ions, or equimolal TrisHCl and Tris (Tris buffer) in aqueous NaCl. The presence of H^+^ in the first set of solutions (at a molality of 0.1 mol kg^–1^) is sufficient to entirely suppress the dissociation of TrisH^+^. In the buffer solutions, the H^+^ molality is controlled by the dissociation of the conjugate acid of Tris, TrisH^+^,^9^ which yields a slightly alkaline solution. The potential, *E* (V), of Cell A is given by the following expression:

1 $$E=E^{0}-\left(\mathrm{RT}/F\right)\cdot \ln\left(aH^{+}\cdot a{\mathrm{Cl}}^{-}\right)$$

where *E* ^0^ (V) is the standard potential of the cell at the temperature *T* (K) of interest, *R* (8.31446 J mol^–1^ K^–1^) is the gas constant, *F* (96 485.332 C mol^–1^) is Faraday’s constant, and prefix *a* denotes activity. The activity product of the H^+^ and Cl^–^ ions can also be written *m*H^+^·*m*Cl^–^·γ_HCl_^2^, where the prefix *m* indicates molality and γ_HCl_ is the mean activity coefficient of H^+^ and Cl^–^ ions in the solution.

A schematic of the Harned cell (Cell A) used at NMIJ is shown in Figure 1 of Maksimov et al.^7^ A flow of dry hydrogen gas at a rate of 4 cm^3^ min^–1^ first passes through a set of three presaturators all of which contain an aqueous solution of the same composition as that being measured. The gas flow next passes into the half-cell of the U-shaped measurement compartment containing the platinum hydrogen electrode and bubbles through the solution. The gas exits the cell via a hydraulic trap designed to prevent any direct contact with the air. This half-cell is connected, with a glass capillary tube, to the other half-cell which contains the same solution and the reference silver–silver chloride electrode. A set of six Harned cells is used for each measurement run. The cells are immersed in a water bath for temperature control.

A total of 18 Harned cells and 18 reference electrodes, belonging to two separate sets, were used in this study. The 12 electrodes used for measurements carried out in 2017 were the same as in our previous study,^7^ and a further six were used for measurements made in 2023. The preparation of hydrogen and reference electrodes is described by Bates,^10^ and the specific procedures used at NMIJ are summarized in the Supporting Information to Maksimov et al.^7^ The ancillary equipment used (for temperature control, and measurement of pressure and potential) is also listed by Maksimov et al., and the setup for Harned cell measurements at NMIJ is described in detail by Ohata.^11^

### 2.1 Solution Compositions and Preparation

The molal ionic strengths (*I*) of the HCl–NaCl–TrisHCl aqueous solutions range from 1.0 to 5.5 mol kg^–1^, with Na^+^ cation fractions *y*Na^+^ (equal to *m*Na^+^/(*m*Na^+^ + *m*TrisH^+^)) of 0.3, 0.5 and 0.7, and a constant H^+^ molality of 0.1 mol kg^–1^. The Tris buffer solutions contain stoichiometric molalities of 0.04 mol kg^–1^ Tris and TrisH^+^ cation (the product of half-neutralization of Tris by HCl) in an NaCl medium with ionic strengths of 0.2, 1.0, and 4.0 mol kg^–1^. The measurement of a wide range of ionic strengths should enable unknown Pitzer model interaction parameters for this mixture to be determined accurately.

The chemicals used in the preparation of the solutions are listed in Table 1. The solid Tris, the purity of which was determined by acidimetric coulometric titration, was stored at room temperature and used directly from sealed bottles without additional drying. The concentrated HCl was diluted with ultrapure water to produce stock solutions of lower concentrations, and their exact molalities (3.6654 ± 0.0014, 3.6603 ± 0.0018, 5.7700 ± 0.0035, and 7.4143 ± 0.0044 mol kg^–1^) were determined by coulometric titration. The purity of the NaCl reagent was determined by argentometric coulometric titration. The salt was dried at 450 °C for 2 h and cooled to room temperature in a desiccator with silica gel before the preparation of NaCl stock solutions of molalities 3.0000 ± 0.0031, 5.1025 ± 0.0031, and 5.1279 ± 0.0031 mol kg^–1^. The concentration uncertainties were calculated from the uncertainty of NaCl purity and the uncertainty of gravimetric preparation.

**Table 1:** Chemicals Used in This Study

chemical	CAS registry #	molar mass (g)	supplier or source	notes
Tris^a^	77-86-1	121.135	FUJIFILM WAKO Pure Chemical Corp.	used as NMIJ CRM 3012-a, purity 99.99 ± 0.10%(*k* = 2) determined by acidimetric coulometric titration
HCl	7647-01-0	36.4609	Kanto Chemical Co.	ultrapure grade aqueous HCl of 31.4 mass % (diluted with water and then molality determined before use)
H_2_O	7732-18-5	18.0153	Milli-Q Ultrapure Water System (Merck)	resistivity 18.2 MΩ cm at 25 °C
NaCl	7647-14-5	58.4430	FUJIFILM WAKO Pure Chemical Corp.	used as NMIJ CRM 3008-a, purity 100.000 ± 0.047%(*k* = 2)determined by argentometric coulometric titration

a 2-Amino-2-(hydroxymethyl)­propane-1,3-diol, C_4_H_11_NO_3_.

All of the studied solutions were prepared gravimetrically as weights in air of HCl and NaCl stock solution aliquots, solid Tris, and water. Buoyancy corrections were carried out using equations presented in Dickson et al.,^12^ and assuming a laboratory temperature of 20 °C. A density of solid Tris of 1.328 g cm^–3^ (typical of those quoted by chemical suppliers) was adopted for the calculation of the buoyancy correction. Densities of aqueous HCl and NaCl solutions were taken from Clegg and Wexler,^13^ and those of water from Kell.^14^ All of the measured solutions were prepared in duplicate.

The standard potentials of the Harned cells are determined from measurements of ∼0.01 mol kg^–1^ HCl at each temperature. The preparation of the dilute aqueous HCl solutions used for measurements carried out in 2017 is described in Section 2.1 of Maksimov.^7^ For the measurements carried out in 2023, the ∼0.1 mol kg^–1^ HCl stock solution was gravimetrically diluted in the same way to obtain the required 0.01 mol kg^–1^ solutions (0.01000012 and 0.00999946 mol kg^–1^ in this work).

### 2.2 Measurements

Cell potentials were measured from 5 to 40 °C for all solutions. Identifiers for the individual cells used, the chloride or HCl molalities of the solutions, and the dates of measurement are listed in Table 2. As can be seen, the measurements for the acidic solutions were carried out in two groups: 3.5–5.5 mol kg^–1^ ionic strength solutions in 2017, and the lower molality solutions in 2023 (and the associated HCl solutions for the determination of standard potentials in 2022 and 2023). All measurements of the 0.04 mol kg^–1^ Tris buffers in aqueous NaCl were made in 2017.

**Table 2:** Cell Identifiers and Dates of Measurements

cells^a^	*m*Cl^–^ (mol kg^–1^)	date	cells	*m*HCl (mol kg^–1^)	date
73–74	0.2 (Tris buffer)^b^	11/10/17	A–F	0.01	21/08/17
75–76	1.0 (Tris buffer)^b^	11/10/17	G–L	0.01	24/08/17
77–78	4.0 (Tris buffer)^b^	11/10/17	M–R	0.01	25/09/17
79–84	3.5	13/11/17	S–X	0.01	23/10/17
85–90	4.0	20/11/17	A1–F1	0.01	14/12/17
91–96	4.5	27/11/17	G1–L1	0.01	06/06/22
97–102	5.0	05/12/17	M1–R1	0.01	23/01/23
103–108	5.5	11/12/17			
1–6	1.0	20/02/23			
7–12	1.5	27/02/23			
13–18	2.0	06/03/23			

a Cells 1–18 are different from those in our previous study that have the same numbers.^7^

b These are the solutions containing equimolal Tris and TrisHCl in aqueous NaCl.

The Harned cells at NMIJ are routinely used for the certification of buffer solutions of ionic strengths up to 0.1 mol kg^–1^, and the measurements in this and our previous study presented some additional difficulties. One of these is related to the fact that the solubility of AgCl increases in solutions containing high concentrations of chloride ions.^15^ Gradual degradation of the reference electrodes due to the dissolution of the electrodeposited layer of silver chloride eventually results in irreversible damage, and it was necessary to measure the solutions in a relatively short space of time. The measurements of solutions made in 2023 (HCl–NaCl–TrisHCl solutions with ionic strengths 1.0–2.0 mol kg^–1^) were made with a comparatively old set of electrodes having in addition a relatively small size of the original silver bulb. The quicker deterioration of those electrodes appears to have caused an offset in the measured cell potentials. This is discussed in Section 4.1, and further details are given in the Supporting Information. Also, during the measurements of some of the most concentrated solutions at the highest temperatures, salt deposition occurred in the first of the three H_2_ presaturator tubes due to loss of water to the dry H_2_ gas stream (see the Supporting Information). These measurements, 11 in total, were discarded.

In dilute buffer solutions, the criterion of stability of cell potential is a voltage drift not exceeding 10 μV h^–1^. For the HCl–NaCl–TrisHCl solutions we observed a similar bias at the lower temperatures 5, 10, 15, and 20 °C, with the drift rising to 50 μV h^–1^ at 30 °C and to 90 μV h^–1^ at 40 °C. Compared to our previous study with HCl–TrisHCl solutions,^7^ the increase at the two last temperatures may have been caused by a stronger impact (on the recorded voltage) of water evaporation from the more concentrated media. This is despite the partial compensation from the preceding set of three presaturators. For the 0.04 mol kg^–1^ Tris buffer in NaCl solutions, the voltage drift was within 10 μV h^–1^, rising slightly to 20 μV h^–1^ at the final temperature of 40 °C.

## 3 Treatment of the Data

The measured cell potentials, *E*_meas_, at the ambient H_2_ partial pressure in the cell are corrected to *p*H_2_ equal to 1 atm using the following relationship:^10^

2 $$E\left(pH_{2},1\mathrm{atm}\right)=E_{\mathrm{meas}}-\mathrm{RT}/\left(2F\right)\cdot \ln\left(pH_{2}\right)$$

where

3 $$pH_{2}=P-pH_{2}O-p\mathrm{HCl}+0.4\cdot \rho \cdot h\cdot g\cdot C$$

*P* (atm) is atmospheric pressure at the time of the measurement, and *p*H_2_O (atm) and *p*HCl (atm) are the equilibrium partial pressures of water and of HCl, respectively, above the solution at the temperature of the measurement. The final term in eq 3 is a further correction in which 0.4 is an empirical factor,^16^ ρ (g cm^–3^) is the density of the solution, *h* (mm) is the depth of immersion of the H_2_ electrode, *g* (9.81 m s^–2^) is the gravitational constant, and *C* (1/101 325 atm Pa^–1^) is a conversion factor from Pa to atm. The influences of the different terms in eq 3 on the adjustment to the measured potentials are given in Table 4 of Maksimov et al.^7^ The contribution of *p*HCl is very small at all temperatures (its maximum calculated value for the acidic solutions measured in this study is 1.9 × 10^–6^ atm). The values of *p*H_2_O are equal to *a*H_2_O·*p* ^o^(H_2_O), neglecting the small difference between partial pressure and fugacity, where *a*H_2_O is the water activity of the solution and *p* ^o^(H_2_O) (atm) the vapor pressure of pure water at the temperature of the measurement. The estimation of *p*HCl, *a*H_2_O, and ρ and their associated uncertainties is summarized in the Supporting Information.

### 3.1 Standard Potentials

Standard potentials, *E* ^0^, of Cell A at each temperature were obtained from the measurements of 0.01 mol kg^–1^ HCl solutions, adjusted to 1 atm *p*H_2_, together with mean activity coefficients of HCl listed by Bates and Robinson.^17^ The effects of the very small deviations of the solution compositions from exactly 0.01 mol kg^–1^ were compensated for by adjusting the potentials *E* as described in Section 3.1 of Maksimov et al.^7^ Information concerning the cells used to determine the standard potentials at each temperature, and the values of *E* ^0^ (with uncertainties) determined in this study, can be found in the Supporting Information. The standard potentials of the cells used for measurements made in 2017 are the same as those presented by Maksimov et al.^7^

The potentials of the measurement solutions, after adjustment to 1 atm *p*H_2_, were further adjusted as described by Maksimov et al.^7^ to be consistent with the standard potentials of Bates and Bower^18^ (column 7 of their Table 1) for ease of comparability. It is these adjusted potentials, *E*(adj.), that are tabulated in this work.

### 3.2 Uncertainties

The overall uncertainty of the measured potential is dominated by that of the voltage measurement. In comparison with our previous study, we estimate that the contribution of the uncertainty of the water activity *u*(*a*H_2_O) to the total, for the most concentrated HCl–NaCl–TrisHCl solutions at the highest temperatures, was greater by about 2 orders of magnitude but was still equal to only ∼0.6% of the total uncertainty of the cell potential. The uncertainty of the voltage measurement was calculated as a combined value of cell potential drift (see Section 2.2 for its numeric values) at the experimental temperature and the standard deviation (SD) of two duplicate measurements:

4 $$u\left(E\right)={\left[{\left(\mathrm{drift}\right)}^{2}+{\left(\mathrm{SD}\right)}^{2}]\right)}^{1/2}$$

In general, the *u*(*E*) values for the HCl–NaCl–TrisHCl solutions were found to be larger than the uncertainties of the measurements in our previous study. They increase with temperature, reaching quite a significant value of 200 μV at 40 °C for solutions with ionic strengths of 3.5 mol kg^–1^ and above. In addition to the more difficult nature of the measurand, slight differences in the geometrical design of duplicate cells (the diameter of the inner capillary tubes, especially) seem likely to be an important influence on the repeatability of measurements for concentrated solutions at temperatures above 25 °C due to water evaporation and transfer.

The *u*(*E*) for 0.04 mol kg^–1^ Tris buffer in a NaCl medium was approximately 100 μV for all studied temperatures.

## 4 Results and Discussion

In this section, we discuss the results for the two types of solutions and compare them with existing literature data and with model calculations.

### 4.1 Aqueous HCl–NaCl–TrisHCl Solutions

Measured cell potentials, corrected to *p*H_2_ equal to 1 atm and adjusted to be consistent with the standard potentials of Bates and Bower,^18^ are listed in Tables 3 and 4 together with values of γ_HCl_ determined from the data. In Tables S6 and S7 of the Supporting Information the original measured potentials are listed, together with other information needed in eqs 2 and 3 to adjust the data to 1 atm *p*H_2_, and also the estimated uncertainties in γ_HCl_.

**Table 3:** Harned Cell Results for HCl–NaCl–TrisHCl Solutions at Ionic Strengths of 3.5 mol kg^–1^ and above, Including Calculated Mean Activity Coefficients of HCl^a^

*t* (°C)	*m*Cl^–^ (mol kg^–1^)	*y*Na^+^	*m*HCl (mol kg^–1^)	*m*TrisHCl (mol kg^–1^)	*m*NaCl (mol kg^–1^)	*E*(adj.)^b^ (V)	*u*(*E*)^b^ (mV)	γ_HCl_		*t* (°C)	*m*Cl^–^ (mol kg^–1^)	*y*Na^+^	*m*HCl (mol kg^–1^)	*m*TrisHCl (mol kg^–1^)	*m*NaCl (mol kg^–1^)	*E*(adj.)^b^ (V)	*u*(*E*)^b^ (mV)	γ_HCl_
5	3.5	0.30	0.10012	2.37995	1.02003	0.26089	0.130	0.9656		20	4.5	0.30	0.10026	3.07973	1.32007	0.24370	0.011	1.0399
5	3.5	0.30	0.10011	2.37985	1.02004	0.26071	0.130	0.9693		20	4.5	0.30	0.10020	3.07987	1.32003	0.24370	0.011	1.0403
5	3.5	0.50	0.10005	1.70001	1.70007	0.25762	0.011	1.0342		20	4.5	0.50	0.10014	2.19992	2.20003	0.23878	0.011	1.1470
5	3.5	0.50	0.10007	1.69994	1.70007	0.25762	0.011	1.0341		20	4.5	0.50	0.10018	2.19982	2.20002	0.23878	0.011	1.1466
5	3.5	0.70	0.10002	1.01997	2.38012	0.25423	0.075	1.1101		20	4.5	0.70	0.10010	1.31994	3.08015	0.23329	0.011	1.2788
5	3.5	0.70	0.10007	1.01992	2.38007	0.25413	0.075	1.1122		20	4.5	0.70	0.10012	1.31984	3.08009	0.23329	0.011	1.2788
10	3.5	0.30	0.10012	2.37995	1.02003	0.25925	0.140	0.9547		25	4.5	0.30	0.10026	3.07973	1.32007	0.24170	0.020	1.0226
10	3.5	0.30	0.10011	2.37985	1.02004	0.25905	0.140	0.9586		25	4.5	0.30	0.10020	3.07987	1.32003	0.24170	0.020	1.0230
10	3.5	0.50	0.10005	1.70001	1.70007	0.25585	0.011	1.0239		25	4.5	0.50	0.10014	2.19992	2.20003	0.23668	0.020	1.1283
10	3.5	0.50	0.10007	1.69994	1.70007	0.25586	0.011	1.0236		25	4.5	0.50	0.10018	2.19982	2.20002	0.23668	0.020	1.1279
10	3.5	0.70	0.10002	1.01997	2.38012	0.25234	0.012	1.1004		25	4.5	0.70	0.10010	1.31994	3.08015	0.23104	0.020	1.2594
10	3.5	0.70	0.10007	1.01992	2.38007	0.25233	0.012	1.1003		25	4.5	0.70	0.10012	1.31984	3.08009	0.23104	0.020	1.2592
15	3.5	0.30	0.10012	2.37995	1.02003	0.25755	0.140	0.9423		30	4.5	0.30	0.10026	3.07973	1.32007	0.23957	0.050	1.0062
15	3.5	0.30	0.10011	2.37985	1.02004	0.25735	0.140	0.9462		30	4.5	0.30	0.10020	3.07987	1.32003	0.23957	0.050	1.0065
15	3.5	0.50	0.10005	1.70001	1.70007	0.25404	0.011	1.0116		30	4.5	0.50	0.10014	2.19992	2.20003	0.23446	0.050	1.1102
15	3.5	0.50	0.10007	1.69994	1.70007	0.25405	0.011	1.0114		30	4.5	0.50	0.10018	2.19982	2.20002	0.23446	0.050	1.1099
15	3.5	0.70	0.10002	1.01997	2.38012	0.25042	0.011	1.0883		30	4.5	0.70	0.10012	1.31984	3.08009	0.22880	0.180	1.2373
15	3.5	0.70	0.10007	1.01992	2.38007	0.25041	0.011	1.0882		40	4.5	0.30	0.10026	3.07973	1.32007	0.23513	0.200	0.9710
20	3.5	0.30	0.10012	2.37995	1.02003	0.25569	0.140	0.9306		40	4.5	0.50	0.10014	2.19992	2.20003	0.22987	0.090	1.0712
20	3.5	0.30	0.10011	2.37985	1.02004	0.25549	0.140	0.9344		40	4.5	0.50	0.10018	2.19982	2.20002	0.22988	0.090	1.0708
20	3.5	0.50	0.10005	1.70001	1.70007	0.25211	0.011	0.9993		40	4.5	0.70	0.10012	1.31984	3.08009	0.22405	0.200	1.1932
20	3.5	0.50	0.10007	1.69994	1.70007	0.25212	0.011	0.9991		5	5.0	0.30	0.10035	3.42967	1.47001	0.24328	0.076	1.1653
20	3.5	0.70	0.10002	1.01997	2.38012	0.24835	0.011	1.0766		5	5.0	0.30	0.10032	3.42990	1.47001	0.24317	0.076	1.1680
20	3.5	0.70	0.10007	1.01992	2.38007	0.24835	0.011	1.0764		5	5.0	0.50	0.10021	2.44982	2.45002	0.23792	0.010	1.3039
25	3.5	0.30	0.10012	2.37995	1.02003	0.25377	0.150	0.9174		5	5.0	0.50	0.10024	2.44979	2.45005	0.23792	0.010	1.3037
25	3.5	0.30	0.10011	2.37985	1.02004	0.25356	0.150	0.9212		5	5.0	0.70	0.10014	1.46982	3.42988	0.23178	0.010	1.4826
25	3.5	0.50	0.10005	1.70001	1.70007	0.25011	0.021	0.9856		5	5.0	0.70	0.10013	1.46993	3.43008	0.23179	0.010	1.4825
25	3.5	0.50	0.10007	1.69994	1.70007	0.25011	0.021	0.9853		10	5.0	0.30	0.10035	3.42967	1.47001	0.24146	0.093	1.1488
25	3.5	0.70	0.10002	1.01997	2.38012	0.24621	0.020	1.0633		10	5.0	0.30	0.10032	3.42990	1.47001	0.24133	0.093	1.1520
25	3.5	0.70	0.10007	1.01992	2.38007	0.24621	0.020	1.0630		10	5.0	0.50	0.10021	2.44982	2.45002	0.23600	0.010	1.2857
30	3.5	0.30	0.10012	2.37995	1.02003	0.25170	0.140	0.9052		10	5.0	0.50	0.10024	2.44979	2.45005	0.23600	0.010	1.2855
30	3.5	0.30	0.10011	2.37985	1.02004	0.25151	0.140	0.9085		10	5.0	0.70	0.10014	1.46982	3.42988	0.22977	0.010	1.4613
30	3.5	0.50	0.10005	1.70001	1.70007	0.24797	0.050	0.9725		10	5.0	0.70	0.10013	1.46993	3.43008	0.22977	0.010	1.4613
30	3.5	0.50	0.10007	1.69994	1.70007	0.24798	0.050	0.9723		15	5.0	0.30	0.10035	3.42967	1.47001	0.23961	0.110	1.1300
30	3.5	0.70	0.10002	1.01997	2.38012	0.24393	0.050	1.0507		15	5.0	0.30	0.10032	3.42990	1.47001	0.23946	0.110	1.1336
30	3.5	0.70	0.10007	1.01992	2.38007	0.24394	0.050	1.0503		15	5.0	0.50	0.10021	2.44982	2.45002	0.23405	0.010	1.2650
40	3.5	0.30	0.10012	2.37995	1.02003	0.24733	0.200	0.8790		15	5.0	0.50	0.10024	2.44979	2.45005	0.23405	0.010	1.2648
40	3.5	0.30	0.10011	2.37985	1.02004	0.24707	0.200	0.8832		15	5.0	0.70	0.10014	1.46982	3.42988	0.22772	0.010	1.4374
40	3.5	0.50	0.10005	1.70001	1.70007	0.24354	0.090	0.9432		15	5.0	0.70	0.10013	1.46993	3.43008	0.22772	0.010	1.4374
40	3.5	0.50	0.10007	1.69994	1.70007	0.24354	0.090	0.9431		20	5.0	0.30	0.10035	3.42967	1.47001	0.23767	0.012	1.1112
40	3.5	0.70	0.10002	1.01997	2.38012	0.23906	0.250	1.0251		20	5.0	0.30	0.10032	3.42990	1.47001	0.23766	0.012	1.1115
40	3.5	0.70	0.10007	1.01992	2.38007	0.23939	0.250	1.0185		20	5.0	0.50	0.10021	2.44982	2.45002	0.23202	0.010	1.2433
5	4.0	0.30	0.10022	2.72986	1.17003	0.25494	0.088	1.0222		20	5.0	0.50	0.10024	2.44979	2.45005	0.23202	0.010	1.2431
5	4.0	0.30	0.10021	2.72974	1.16997	0.25481	0.088	1.0250		20	5.0	0.70	0.10014	1.46982	3.42988	0.22559	0.010	1.4127
5	4.0	0.50	0.10016	1.94991	1.95005	0.25098	0.012	1.1106		20	5.0	0.70	0.10013	1.46993	3.43008	0.22559	0.010	1.4127
5	4.0	0.50	0.10016	1.94975	1.94994	0.25099	0.012	1.1104		25	5.0	0.30	0.10035	3.42967	1.47001	0.23562	0.021	1.0914
5	4.0	0.70	0.10006	1.16994	2.73009	0.24657	0.074	1.2182		25	5.0	0.30	0.10032	3.42990	1.47001	0.23562	0.021	1.0917
5	4.0	0.70	0.10008	1.16994	2.73009	0.24667	0.074	1.2154		25	5.0	0.50	0.10021	2.44982	2.45002	0.22988	0.020	1.2213
10	4.0	0.30	0.10022	2.72986	1.17003	0.25323	0.099	1.0097		25	5.0	0.50	0.10024	2.44979	2.45005	0.22988	0.020	1.2211
10	4.0	0.30	0.10021	2.72974	1.16997	0.25309	0.099	1.0127		25	5.0	0.70	0.10014	1.46982	3.42988	0.22321	0.140	1.3911
10	4.0	0.50	0.10016	1.94991	1.95005	0.24916	0.012	1.0978		25	5.0	0.70	0.10013	1.46993	3.43008	0.22340	0.140	1.3859
10	4.0	0.50	0.10016	1.94975	1.94994	0.24917	0.012	1.0977		30	5.0	0.30	0.10035	3.42967	1.47001	0.23343	0.050	1.0731
10	4.0	0.70	0.10006	1.16994	2.73009	0.24464	0.076	1.2052		30	5.0	0.30	0.10032	3.42990	1.47001	0.23343	0.050	1.0733
10	4.0	0.70	0.10008	1.16994	2.73009	0.24474	0.076	1.2024		30	5.0	0.50	0.10021	2.44982	2.45002	0.22762	0.050	1.2001
15	4.0	0.30	0.10022	2.72986	1.17003	0.25148	0.130	0.9955		30	5.0	0.50	0.10024	2.44979	2.45005	0.22763	0.050	1.1999
15	4.0	0.30	0.10021	2.72974	1.16997	0.25129	0.130	0.9993		30	5.0	0.70	0.10013	1.46993	3.43008	0.22110	0.180	1.3603
15	4.0	0.50	0.10016	1.94991	1.95005	0.24731	0.012	1.0831		40	5.0	0.30	0.10035	3.42967	1.47001	0.22889	0.200	1.0338
15	4.0	0.50	0.10016	1.94975	1.94994	0.24732	0.012	1.0830		40	5.0	0.50	0.10021	2.44982	2.45002	0.22293	0.090	1.1552
15	4.0	0.70	0.10006	1.16994	2.73009	0.24265	0.086	1.1902		40	5.0	0.50	0.10024	2.44979	2.45005	0.22294	0.090	1.1549
15	4.0	0.70	0.10008	1.16994	2.73009	0.24277	0.086	1.1872		40	5.0	0.70	0.10013	1.46993	3.43008	0.21633	0.200	1.3061
20	4.0	0.30	0.10022	2.72986	1.17003	0.24960	0.130	0.9817		5	5.5	0.30	0.10034	3.77981	1.61999	0.23815	0.150	1.2365
20	4.0	0.30	0.10021	2.72974	1.16997	0.24941	0.130	0.9854		5	5.5	0.30	0.10010	3.77057	1.61614	0.23836	0.150	1.2340
20	4.0	0.50	0.10016	1.94991	1.95005	0.24535	0.011	1.0681		5	5.5	0.50	0.10026	2.69965	2.70002	0.23202	0.160	1.4057
20	4.0	0.50	0.10016	1.94975	1.94994	0.24535	0.011	1.0681		5	5.5	0.50	0.10027	2.69969	2.70005	0.23224	0.160	1.3991
20	4.0	0.70	0.10006	1.16994	2.73009	0.24059	0.085	1.1742		5	5.5	0.70	0.10016	1.61984	3.78003	0.22509	0.150	1.6253
20	4.0	0.70	0.10008	1.16994	2.73009	0.24070	0.085	1.1714		5	5.5	0.70	0.10015	1.61985	3.77996	0.22488	0.150	1.6325
25	4.0	0.30	0.10022	2.72986	1.17003	0.24762	0.120	0.9667		10	5.5	0.30	0.10034	3.77981	1.61999	0.23631	0.150	1.2173
25	4.0	0.30	0.10021	2.72974	1.16997	0.24745	0.120	0.9701		10	5.5	0.30	0.10010	3.77057	1.61614	0.23652	0.150	1.2150
25	4.0	0.50	0.10016	1.94991	1.95005	0.24327	0.020	1.0525		10	5.5	0.50	0.10026	2.69965	2.70002	0.23007	0.150	1.3837
25	4.0	0.50	0.10016	1.94975	1.94994	0.24327	0.020	1.0525		10	5.5	0.50	0.10027	2.69969	2.70005	0.23029	0.150	1.3775
25	4.0	0.70	0.10006	1.16994	2.73009	0.23841	0.100	1.1576		10	5.5	0.70	0.10016	1.61984	3.78003	0.22303	0.130	1.5994
25	4.0	0.70	0.10008	1.16994	2.73009	0.23854	0.100	1.1544		10	5.5	0.70	0.10015	1.61985	3.77996	0.22285	0.130	1.6055
30	4.0	0.30	0.10022	2.72986	1.17003	0.24551	0.120	0.9527		15	5.5	0.30	0.10034	3.77981	1.61999	0.23443	0.130	1.1959
30	4.0	0.30	0.10021	2.72974	1.16997	0.24536	0.120	0.9555		15	5.5	0.30	0.10010	3.77057	1.61614	0.23462	0.130	1.1944
30	4.0	0.50	0.10016	1.94991	1.95005	0.24107	0.050	1.0374		15	5.5	0.50	0.10026	2.69965	2.70002	0.22811	0.140	1.3590
30	4.0	0.50	0.10016	1.94975	1.94994	0.24108	0.050	1.0374		15	5.5	0.50	0.10027	2.69969	2.70005	0.22831	0.140	1.3535
30	4.0	0.70	0.10006	1.16994	2.73009	0.23598	0.200	1.1443		15	5.5	0.70	0.10016	1.61984	3.78003	0.22095	0.085	1.5703
30	4.0	0.70	0.10008	1.16994	2.73009	0.23625	0.200	1.1382		15	5.5	0.70	0.10015	1.61985	3.77996	0.22084	0.085	1.5742
40	4.0	0.30	0.10022	2.72986	1.17003	0.24110	0.170	0.9223		20	5.5	0.30	0.10034	3.77981	1.61999	0.23247	0.074	1.1742
40	4.0	0.30	0.10021	2.72974	1.16997	0.24090	0.170	0.9259		20	5.5	0.30	0.10010	3.77057	1.61614	0.23258	0.074	1.1746
40	4.0	0.50	0.10016	1.94991	1.95005	0.23653	0.200	1.0041		20	5.5	0.50	0.10026	2.69965	2.70002	0.22606	0.120	1.3337
40	4.0	0.70	0.10008	1.16994	2.73009	0.23159	0.200	1.1009		20	5.5	0.50	0.10027	2.69969	2.70005	0.22623	0.120	1.3292
5	4.5	0.30	0.10026	3.07973	1.32007	0.24919	0.110	1.0863		20	5.5	0.70	0.10016	1.61984	3.78003	0.21881	0.011	1.5403
5	4.5	0.30	0.10020	3.07987	1.32003	0.24904	0.110	1.0901		20	5.5	0.70	0.10015	1.61985	3.77996	0.21880	0.011	1.5405
5	4.5	0.50	0.10014	2.19992	2.20003	0.24454	0.073	1.1976		25	5.5	0.30	0.10034	3.77981	1.61999	0.23040	0.079	1.1520
5	4.5	0.50	0.10018	2.19982	2.20002	0.24464	0.073	1.1948		25	5.5	0.30	0.10010	3.77057	1.61614	0.23051	0.079	1.1524
5	4.5	0.70	0.10010	1.31994	3.08015	0.23935	0.010	1.3348		25	5.5	0.50	0.10026	2.69965	2.70002	0.22389	0.120	1.3081
5	4.5	0.70	0.10012	1.31984	3.08009	0.23935	0.010	1.3347		25	5.5	0.50	0.10027	2.69969	2.70005	0.22406	0.120	1.3039
10	4.5	0.30	0.10026	3.07973	1.32007	0.24743	0.096	1.0719		25	5.5	0.70	0.10016	1.61984	3.78003	0.21642	0.088	1.5137
10	4.5	0.30	0.10020	3.07987	1.32003	0.24730	0.096	1.0752		25	5.5	0.70	0.10015	1.61985	3.77996	0.21654	0.088	1.5102
10	4.5	0.50	0.10014	2.19992	2.20003	0.24270	0.011	1.1817		30	5.5	0.30	0.10034	3.77981	1.61999	0.22824	0.051	1.1301
10	4.5	0.50	0.10018	2.19982	2.20002	0.24271	0.011	1.1813		30	5.5	0.30	0.10010	3.77057	1.61614	0.22825	0.051	1.1326
10	4.5	0.70	0.10010	1.31994	3.08015	0.23737	0.010	1.3183		30	5.5	0.50	0.10026	2.69965	2.70002	0.22159	0.150	1.2840
10	4.5	0.70	0.10012	1.31984	3.08009	0.23737	0.010	1.3182		30	5.5	0.50	0.10027	2.69969	2.70005	0.22179	0.150	1.2790
15	4.5	0.30	0.10026	3.07973	1.32007	0.24562	0.110	1.0559		30	5.5	0.70	0.10015	1.61985	3.77996	0.21429	0.180	1.4773
15	4.5	0.30	0.10020	3.07987	1.32003	0.24547	0.110	1.0593		40	5.5	0.30	0.10034	3.77981	1.61999	0.22377	0.090	1.0838
15	4.5	0.50	0.10014	2.19992	2.20003	0.24078	0.011	1.1647		40	5.5	0.30	0.10010	3.77057	1.61614	0.22377	0.090	1.0864
15	4.5	0.50	0.10018	2.19982	2.20002	0.24079	0.011	1.1644		40	5.5	0.50	0.10026	2.69965	2.70002	0.21690	0.190	1.2313
15	4.5	0.70	0.10010	1.31994	3.08015	0.23537	0.011	1.2991		40	5.5	0.50	0.10027	2.69969	2.70005	0.21715	0.190	1.2257
15	4.5	0.70	0.10012	1.31984	3.08009	0.23536	0.011	1.2991										

a The first nine entries on each row is one set of results, and the second nine entries (also starting with temperature *t*) is a second set. Columns *m*Cl^–^ and *y*Na^+^ contain rounded values, and exact molalities can be calculated from the listed *m*HCl, *m*NaCl and *m*TrisHCl. More complete results, including estimated uncertainties of γ_HCl_, can be found in the Supporting Information.

b Cell potentials and their uncertainties are listed here to a fixed 5 digits and 3 digits following the decimal point, respectively. This was done for simplicity. In some cases (uncertainties of around 0.01 mV) an extra digit in *E*(adj.) is appropriate,^28^ or (for uncertainties of around 0.1 mV or higher) the removal of a final zero (*u*(*E*)).

**Table 4:** Harned Cell Results For HCl–NaCl–TrisHCl Solutions at Ionic Strengths of 2.0 mol kg^–1^ and below, Including Calculated Mean Activity Coefficients of HCl^a^

*t* (°C)	*m*Cl^–^ (mol kg^–1^)	*y*Na^+^	*m*HCl (mol kg^–1^)	*m*TrisHCl (mol kg^–1^)	*m*NaCl (mol kg^–1^)	*E*(adj.)^b^ (V)	*u*(*E*)^b^ (mV)	γ_HCl_		*t* (°C)	*m*Cl^–^ (mol kg^–1^)	*y*Na^+^	*m*HCl (mol kg^–1^)	*m*TrisHCl (mol kg^–1^)	*m*NaCl (mol kg^–1^)	*E*(adj.)^b^ (V)	*u*(*E*)^b^ (mV)	γ_HCl_
5	1.0	0.30	0.10000	0.62997	0.26993	0.30334	0.012	0.7457		25	1.5	0.30	0.09990	0.98005	0.41996	0.28578	0.100	0.7525
5	1.0	0.30	0.10001	0.63004	0.26995	0.30335	0.012	0.7455		25	1.5	0.30	0.09994	0.98001	0.42001	0.28592	0.100	0.7503
5	1.0	0.50	0.10002	0.44999	0.45001	0.30287	0.013	0.7529		25	1.5	0.50	0.09995	0.69999	0.70001	0.28461	0.021	0.7697
5	1.0	0.50	0.10024	0.44997	0.44998	0.30286	0.013	0.7522		25	1.5	0.50	0.09998	0.69998	0.70002	0.28460	0.021	0.7697
5	1.0	0.70	0.10010	0.27001	0.62991	0.30210	0.011	0.7648		25	1.5	0.70	0.10000	0.42001	0.97997	0.28320	0.020	0.7908
5	1.0	0.70	0.09993	0.27002	0.62997	0.30210	0.011	0.7654		25	1.5	0.70	0.10002	0.42000	0.97998	0.28321	0.020	0.7907
10	1.0	0.30	0.10000	0.62997	0.26993	0.30213	0.072	0.7422		30	1.5	0.30	0.09990	0.98005	0.41996	0.28394	0.120	0.7468
10	1.0	0.30	0.10001	0.63004	0.26995	0.30223	0.072	0.7406		30	1.5	0.30	0.09994	0.98001	0.42001	0.28409	0.120	0.7444
10	1.0	0.50	0.10002	0.44999	0.45001	0.30163	0.073	0.7498		30	1.5	0.50	0.09995	0.69999	0.70001	0.28272	0.050	0.7642
10	1.0	0.50	0.10024	0.44997	0.44998	0.30153	0.073	0.7505		30	1.5	0.50	0.09998	0.69998	0.70002	0.28272	0.050	0.7642
10	1.0	0.70	0.10010	0.27001	0.62991	0.30081	0.010	0.7623		30	1.5	0.70	0.10000	0.42001	0.97997	0.28128	0.050	0.7854
10	1.0	0.70	0.09993	0.27002	0.62997	0.30081	0.010	0.7629		30	1.5	0.70	0.10002	0.42000	0.97998	0.28128	0.050	0.7853
15	1.0	0.30	0.10000	0.62997	0.26993	0.30084	0.077	0.7378		40	1.5	0.30	0.09990	0.98005	0.41996	0.28026	0.140	0.7302
15	1.0	0.30	0.10001	0.63004	0.26995	0.30094	0.077	0.7362		40	1.5	0.30	0.09994	0.98001	0.42001	0.28041	0.140	0.7281
15	1.0	0.50	0.10002	0.44999	0.45001	0.30030	0.013	0.7458		40	1.5	0.50	0.09995	0.69999	0.70001	0.27897	0.090	0.7477
15	1.0	0.50	0.10024	0.44997	0.44998	0.30029	0.013	0.7450		40	1.5	0.50	0.09998	0.69998	0.70002	0.27896	0.090	0.7477
15	1.0	0.70	0.10010	0.27001	0.62991	0.29945	0.010	0.7583		40	1.5	0.70	0.10000	0.42001	0.97997	0.27744	0.090	0.7690
15	1.0	0.70	0.09993	0.27002	0.62997	0.29945	0.010	0.7590		40	1.5	0.70	0.10002	0.42000	0.97998	0.27745	0.090	0.7688
20	1.0	0.30	0.10000	0.62997	0.26993	0.29938	0.085	0.7338		5	2.0	0.30	0.10006	1.33000	0.56996	0.28222	0.140	0.8190
20	1.0	0.30	0.10001	0.63004	0.26995	0.29949	0.085	0.7321		5	2.0	0.30	0.10004	1.32995	0.56999	0.28241	0.140	0.8157
20	1.0	0.50	0.10002	0.44999	0.45001	0.29879	0.012	0.7423		5	2.0	0.50	0.10009	0.94979	0.95026	0.28070	0.160	0.8452
20	1.0	0.50	0.10024	0.44997	0.44998	0.29878	0.012	0.7415		5	2.0	0.50	0.10001	0.95002	0.94994	0.28092	0.160	0.8417
20	1.0	0.70	0.10010	0.27001	0.62991	0.29793	0.010	0.7547		5	2.0	0.70	0.10000	0.57000	1.32998	0.27928	0.140	0.8710
20	1.0	0.70	0.09993	0.27002	0.62997	0.29793	0.010	0.7554		5	2.0	0.70	0.09995	0.57006	1.32999	0.27909	0.140	0.8747
25	1.0	0.30	0.10000	0.62997	0.26993	0.29783	0.082	0.7286		10	2.0	0.30	0.10006	1.33000	0.56996	0.28077	0.140	0.8128
25	1.0	0.30	0.10001	0.63004	0.26995	0.29794	0.082	0.7270		10	2.0	0.30	0.10004	1.32995	0.56999	0.28096	0.140	0.8097
25	1.0	0.50	0.10002	0.44999	0.45001	0.29721	0.021	0.7373		10	2.0	0.50	0.10009	0.94979	0.95026	0.27916	0.160	0.8400
25	1.0	0.50	0.10024	0.44997	0.44998	0.29720	0.021	0.7366		10	2.0	0.50	0.10001	0.95002	0.94994	0.27938	0.160	0.8365
25	1.0	0.70	0.10010	0.27001	0.62991	0.29631	0.020	0.7501		10	2.0	0.70	0.10000	0.57000	1.32998	0.27767	0.140	0.8664
25	1.0	0.70	0.09993	0.27002	0.62997	0.29631	0.020	0.7507		10	2.0	0.70	0.09995	0.57006	1.32999	0.27748	0.140	0.8700
30	1.0	0.30	0.10000	0.62997	0.26993	0.29611	0.097	0.7243		15	2.0	0.30	0.10006	1.33000	0.56996	0.27924	0.140	0.8057
30	1.0	0.30	0.10001	0.63004	0.26995	0.29623	0.097	0.7226		15	2.0	0.30	0.10004	1.32995	0.56999	0.27943	0.140	0.8026
30	1.0	0.50	0.10002	0.44999	0.45001	0.29546	0.050	0.7332		15	2.0	0.50	0.10009	0.94979	0.95026	0.27755	0.170	0.8333
30	1.0	0.50	0.10024	0.44997	0.44998	0.29545	0.050	0.7324		15	2.0	0.50	0.10001	0.95002	0.94994	0.27779	0.170	0.8297
30	1.0	0.70	0.10010	0.27001	0.62991	0.29454	0.050	0.7459		15	2.0	0.70	0.10000	0.57000	1.32998	0.27600	0.140	0.8602
30	1.0	0.70	0.09993	0.27002	0.62997	0.29454	0.050	0.7466		15	2.0	0.70	0.09995	0.57006	1.32999	0.27580	0.140	0.8639
40	1.0	0.30	0.10000	0.62997	0.26993	0.29265	0.120	0.7106		20	2.0	0.30	0.10006	1.33000	0.56996	0.27755	0.150	0.7989
40	1.0	0.30	0.10001	0.63004	0.26995	0.29276	0.120	0.7090		20	2.0	0.30	0.10004	1.32995	0.56999	0.27776	0.150	0.7957
40	1.0	0.50	0.10002	0.44999	0.45001	0.29195	0.120	0.7197		20	2.0	0.50	0.10009	0.94979	0.95026	0.27581	0.170	0.8269
40	1.0	0.50	0.10024	0.44997	0.44998	0.29185	0.120	0.7202		20	2.0	0.50	0.10001	0.95002	0.94994	0.27604	0.170	0.8234
40	1.0	0.70	0.10010	0.27001	0.62991	0.29097	0.090	0.7326		20	2.0	0.70	0.10000	0.57000	1.32998	0.27419	0.150	0.8542
40	1.0	0.70	0.09993	0.27002	0.62997	0.29097	0.090	0.7333		20	2.0	0.70	0.09995	0.57006	1.32999	0.27397	0.150	0.8581
5	1.5	0.30	0.09990	0.98005	0.41996	0.29181	0.110	0.7747		25	2.0	0.30	0.10006	1.33000	0.56996	0.27580	0.020	0.7907
5	1.5	0.30	0.09994	0.98001	0.42001	0.29197	0.110	0.7721		25	2.0	0.30	0.10004	1.32995	0.56999	0.27581	0.020	0.7907
5	1.5	0.50	0.09995	0.69999	0.70001	0.29082	0.011	0.7908		25	2.0	0.50	0.10009	0.94979	0.95026	0.27399	0.170	0.8189
5	1.5	0.50	0.09998	0.69998	0.70002	0.29081	0.011	0.7908		25	2.0	0.50	0.10001	0.95002	0.94994	0.27423	0.170	0.8155
5	1.5	0.70	0.10000	0.42001	0.97997	0.28965	0.010	0.8101		25	2.0	0.70	0.10000	0.57000	1.32998	0.27230	0.150	0.8466
5	1.5	0.70	0.10002	0.42000	0.97998	0.28965	0.010	0.8100		25	2.0	0.70	0.09995	0.57006	1.32999	0.27209	0.150	0.8504
10	1.5	0.30	0.09990	0.98005	0.41996	0.29046	0.110	0.7701		30	2.0	0.30	0.10006	1.33000	0.56996	0.27388	0.160	0.7835
10	1.5	0.30	0.09994	0.98001	0.42001	0.29061	0.110	0.7676		30	2.0	0.30	0.10004	1.32995	0.56999	0.27409	0.160	0.7804
10	1.5	0.50	0.09995	0.69999	0.70001	0.28941	0.011	0.7867		30	2.0	0.50	0.10009	0.94979	0.95026	0.27202	0.180	0.8117
10	1.5	0.50	0.09998	0.69998	0.70002	0.28941	0.011	0.7866		30	2.0	0.50	0.10001	0.95002	0.94994	0.27226	0.180	0.8083
10	1.5	0.70	0.10000	0.42001	0.97997	0.28817	0.011	0.8068		30	2.0	0.70	0.10000	0.57000	1.32998	0.27029	0.170	0.8394
10	1.5	0.70	0.10002	0.42000	0.97998	0.28817	0.011	0.8066		30	2.0	0.70	0.09995	0.57006	1.32999	0.27006	0.170	0.8434
15	1.5	0.30	0.09990	0.98005	0.41996	0.28904	0.110	0.7643		40	2.0	0.30	0.10006	1.33000	0.56996	0.27003	0.190	0.7638
15	1.5	0.30	0.09994	0.98001	0.42001	0.28920	0.110	0.7618		40	2.0	0.30	0.10004	1.32995	0.56999	0.27027	0.190	0.7605
15	1.5	0.50	0.09995	0.69999	0.70001	0.28795	0.011	0.7811		40	2.0	0.50	0.10009	0.94979	0.95026	0.26809	0.200	0.7916
15	1.5	0.50	0.09998	0.69998	0.70002	0.28794	0.011	0.7811		40	2.0	0.50	0.10001	0.95002	0.94994	0.26834	0.200	0.7882
15	1.5	0.70	0.10000	0.42001	0.97997	0.28664	0.011	0.8018		40	2.0	0.70	0.10000	0.57000	1.32998	0.26627	0.200	0.8191
15	1.5	0.70	0.10002	0.42000	0.97998	0.28664	0.011	0.8016		40	2.0	0.70	0.09995	0.57006	1.32999	0.26602	0.200	0.8231
20	1.5	0.30	0.09990	0.98005	0.41996	0.28745	0.120	0.7591										
20	1.5	0.30	0.09994	0.98001	0.42001	0.28762	0.120	0.7564										
20	1.5	0.50	0.09995	0.69999	0.70001	0.28631	0.011	0.7761										
20	1.5	0.50	0.09998	0.69998	0.70002	0.28631	0.011	0.7761										
20	1.5	0.70	0.10000	0.42001	0.97997	0.28497	0.011	0.7967										
20	1.5	0.70	0.10002	0.42000	0.97998	0.28498	0.011	0.7966										

a The first nine entries on each row is one set of results, and the second nine entries (also starting with temperature *t*) is a second set. Columns *m*Cl^–^ and *y*Na^+^ contain rounded values, and exact molalities can be calculated from the listed *m*HCl, *m*NaCl, and *m*TrisHCl. More complete results, including estimated uncertainties of γ_HCl_, can be found in the Supporting Information.

b Cell potentials and their uncertainties are listed here to a fixed 5 digits and 3 digits following the decimal point, respectively. This was done for simplicity. In some cases (uncertainties of around 0.01 mV) an extra digit in *E*(adj.) is appropriate,^28^ or (for uncertainties of around 0.1 mV or higher) the removal of a final zero (*u*(*E*)).

Results at 25 °C are shown in Figure 1, distinguishing the data for each of the three Na^+^ fractions (*y*Na^+^). The solid line represents mean activity coefficients for aqueous HCl–TrisHCl calculated using the same Pitzer model as Maksimov et al.^7^ in their Figure 2b. For compositions corresponding to *y*Na^+^ equal to unity, i.e., aqueous HCl–NaCl, we show model-calculated values at low ionic strengths and also data from several other studies, some of which derive from the application of Harned’s rule to the original measurements (cited in the caption to Figure 1). Our results in Table 4 for low ionic strengths, plotted in the inset of Figure 1, show that measured values (the solid symbols) are not equidistant between the lines for the two end-member cases of *y*Na^+^ equal to zero and one. This appears to suggest that the substitution of TrisH^+^ by Na^+^ in these dilute solutions yields a steep increase in γ_HCl_ that is not apparent in the measurements for the higher ionic strengths, also shown in Figure 1, that were carried out several years before. This behavior is examined below.

**Figure 1:**
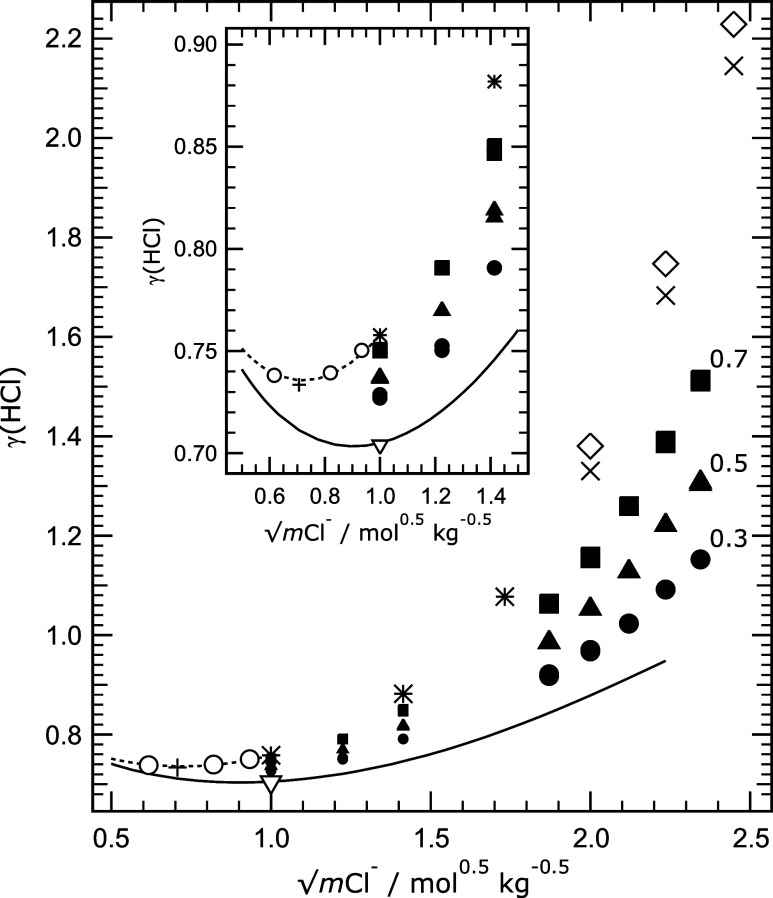
Measured and calculated mean activity coefficients γ_HCl_ (labeled γ­(HCl) for clarity) at 25 °C, plotted against the square root of total Cl^–^ molality (*m*Cl^–^). All data are for solutions containing 0.1 mol kg^–1^ of H^+^. Symbols: dot, solid triangle, and solid square – results of this study at the three Na^+^ cation fractions (*y*Na^+^) indicated on the plot; inverted triangle – measurement from Maksimov et al.^7^ for aqueous HCl–TrisHCl (hence *y*Na^+^ = 0). Other symbols are for aqueous HCl–NaCl solutions (*y*Na^+^ = 1): open circle – Macaskill et al.^22^ (calculated using Harned’s rule coefficients in their Table II); plus – from Harned’s rule coefficients in Table 14-6-2 of Harned and Owen;^23^ asterisk – from Harned’s rule coefficients in Table 1 of Harned;^24^ cross – measurements of Hawkins;^25^ diamond – from Harned’s rule coefficients in Table 4 of Jiang.^26^ Solid symbols at low chloride molalities are reduced in size for clarity. Lines: solid – for aqueous HCl–TrisHCl (*y*Na^+^ = 0) calculated using the Pitzer model of Clegg et al.^6^ including values of parameters θ_H,TrisH_ and ψ_H,TrisH,Cl_ as described in the text; dotted – for aqueous HCl–NaCl calculated using the same Pitzer model. The inset shows the same results up to a 2.0 mol kg^–1^ Cl^–^ molality.

Figure 2, parts a–d, shows values of γ_HCl_ at three different temperatures, as a function of *y*Na^+^ for fixed ionic strengths. At ionic strengths and temperatures for which there are also data for the end-member solutions (*y*Na^+^ equal to zero and one), dotted lines are used to link all the values. The results show that γ_HCl_ is the highest at the lowest temperatures, and the slope with respect to *y*Na^+^ increases with ionic strength.

**Figure 2:**
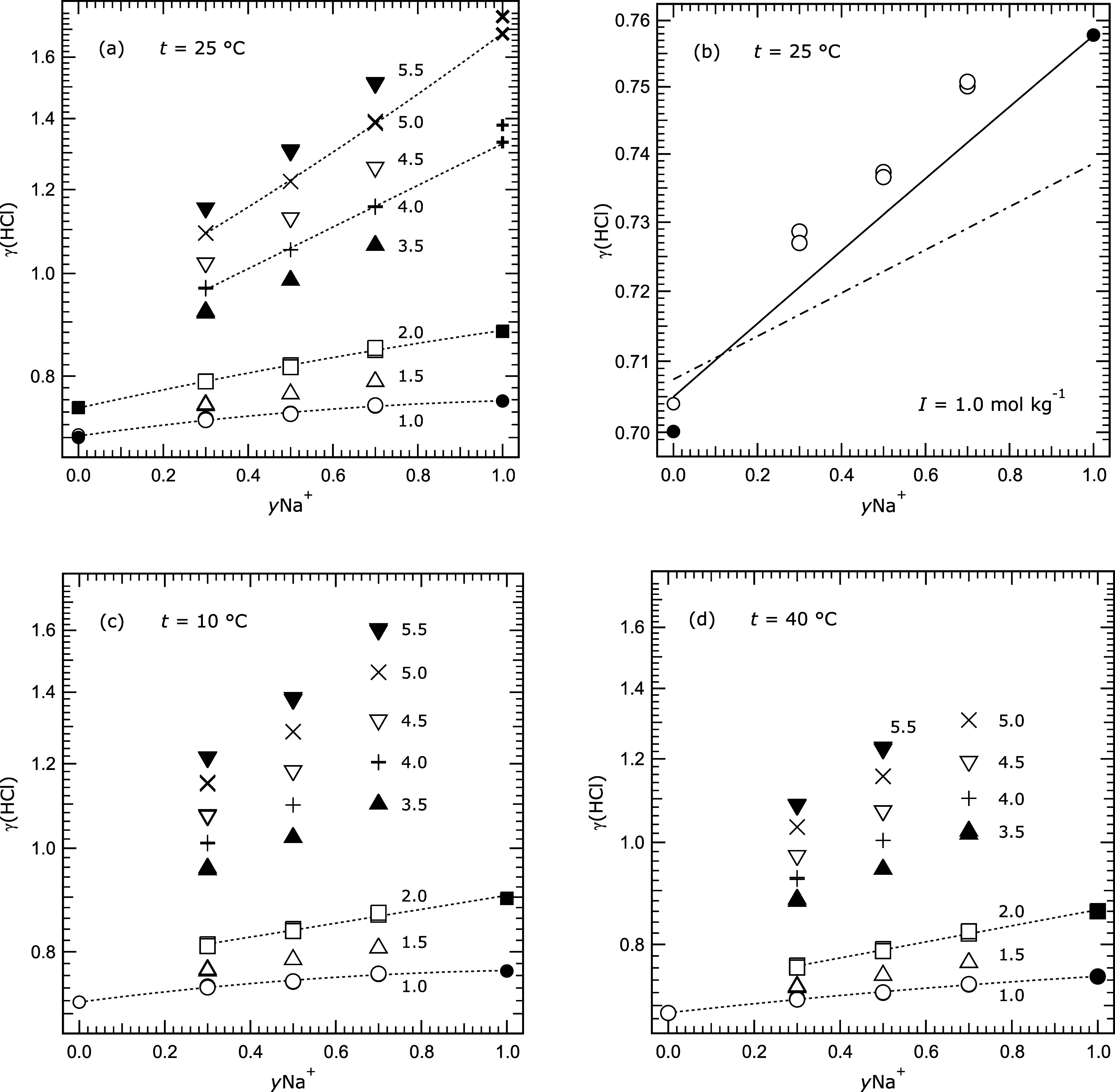
Measured mean activity coefficients γ_HCl_ (labeled γ­(HCl) for clarity) plotted against the Na^+^ cation fraction *y*Na^+^ for different temperatures and ionic strengths as indicated on the plots. Symbols and ionic strengths: dot and open circle – 1.0; triangle – 1.5; square and solid square – 2.0; solid triangle – 3.5; plus – 4.0; inverted triangle – 4.5; cross – 5.0; solid inverted triangle – 5.5 mol kg^–1^. The open circles at *y*Na^+^ = 0 are values from our previous study.^7^ (a) Results at 25 °C, including measurements of Macaskill and Bates^27^ and Bates and Macaskill^19^ (solid symbols at *y*Na^+^ = 0.0), Harned^24^ (solid square and circle at *y*Na^+^ = 1.0), and Hawkins^25^ and Jiang^26^ (bold plus and cross at *y*Na^+^ = 1.0). Dotted lines are visual guides only. (b) Results for ionic strength 1.0 mol kg^–1^ from plot (a). Lines were calculated using the model of Clegg et al.,^6^ as follows: dash-dot – without ternary mixture parameters; solid – with ternary mixture parameters as described in the text. (c, d) Results at 10 and 40 °C, with symbols denoting different ionic strengths as in (a). Data for the other temperatures (5, 15, 20, and 30 °C) are similar and are not shown.

In Figure 2b we compare model-calculated γ_HCl_ with measured values at 25 °C and an ionic strength of 1.0 mol kg^–1^ as a typical example of the results at low ionic strengths. The dashed-dotted line was calculated using Pitzer model parameters for H^+^–Cl^–^, TrisH^+^–Cl^–^, and Na^+^–Cl^–^ interactions only. The addition of ternary parameters for H^+^–Na^+^–Cl^–^ interactions (as used by Clegg et al.^6^), and H^+^–TrisH^+^–Cl^–^ interactions (from Bates and Macaskill^19^), can be shown to yield more accurate predictions of both end-member solutions. However, our measured γ_HCl_ for intermediate values of *y*Na^+^ are consistently higher than predicted, by about 0.0075, even using recently determined parameters for Na^+^–TrisH^+^–Cl^–^ interactions (J. Miladinovic, Pers. comm.). This is equivalent to a difference in potential of about 0.5 mV. No plausible values of the ternary mixture parameters in the Pitzer model for these solutions seem able to account for the observed difference, and we attribute it to the condition of the electrodes used for the low ionic strength measurements, as noted in Section 2.2.

Further examination of this behavior in Figure 3a shows, first, that the offset of the measured γ_HCl_ from the predicted values is similar at all three ionic strengths and does not appear to have a relationship with *y*Na^+^. Second, the modeled γ_HCl_ for aqueous HCl–NaCl (i.e., *y*Na^+^ equal to 1.0) at 2.0 mol kg^–1^ ionic strength is lower than the measured value. Calculations for higher ionic strengths (not shown) yield similar behavior, which suggests that this underprediction may represent small errors in the model for H^+^–Na^+^–Cl^–^ interactions, or perhaps those for Na^+^–Cl^–^ (or H^+^–Cl^–^ in the most concentrated solutions). In Figure 3b we show the difference between adjusted values of γ_HCl_ obtained from our measurements (at *y*Na^+^ equal to 0.3, 0.5, and 0.7) and modeled values. The adjustment is equivalent to a change in the measured potential by 0.5 mV, as determined from the data for the 1.0 mol kg^–1^ ionic strength and noted above. There is quite good agreement, in most cases, to within about ±0.002 in γ_HCl_. The fact that the deviations of Δγ_HCl_ increase with *y*Na^+^ for the solutions at 2.0 mol kg^–1^ ionic strength (relative to values at the two lower ionic strengths) is consistent with the small error in the model mentioned above. Past experience suggests that the Δ*E* values that we observe here, related to the condition of the electrodes, are likely to be roughly constant with temperature. If this is so then these data will still be valuable for constraining a future Pitzer model of these solutions.

**Figure 3:**
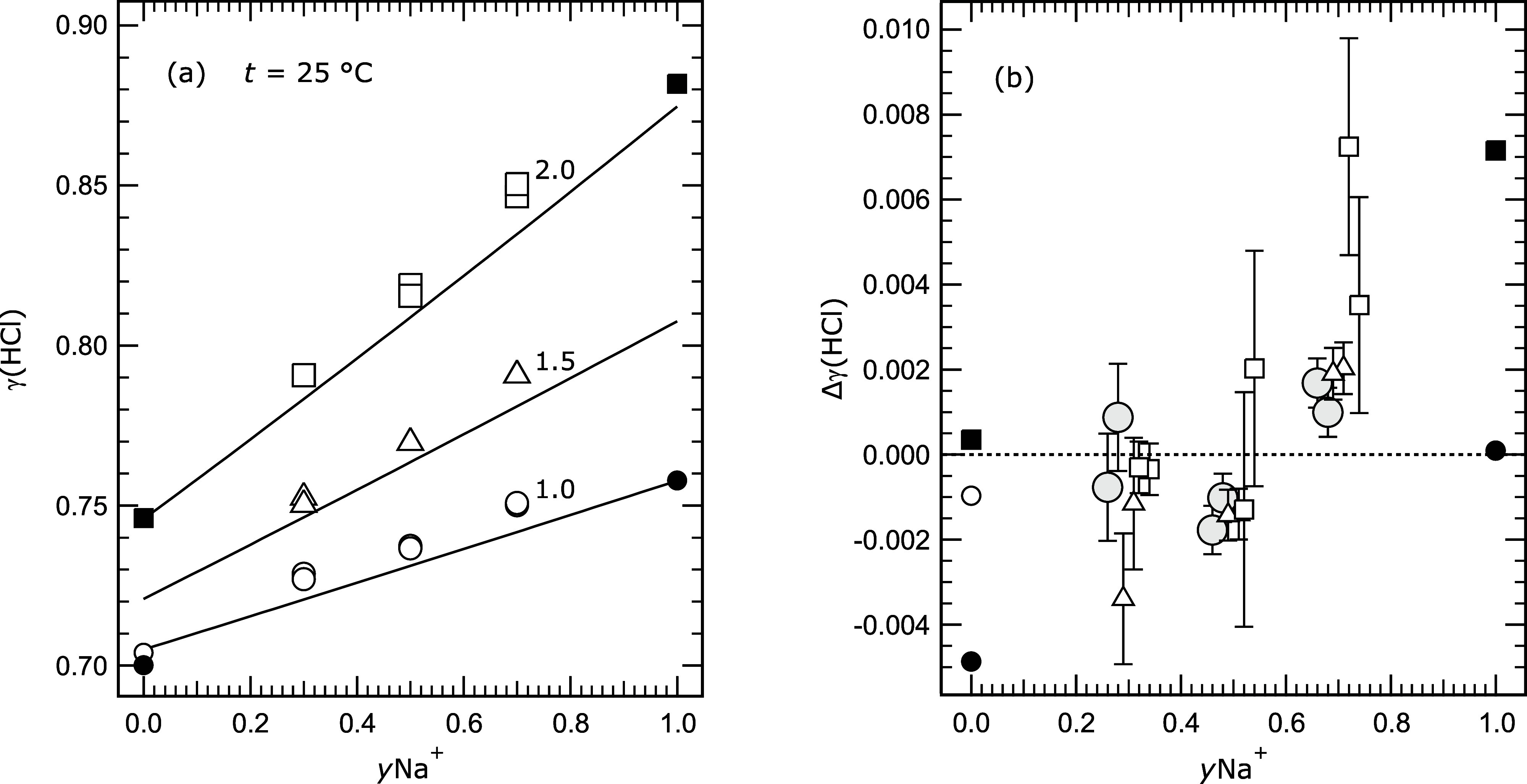
Measured and calculated mean activity coefficients γ_HCl_ (labeled γ­(HCl) for clarity) at 25 °C, for solutions with ionic strengths 1.0, 1.5, and 2.0 mol kg^–1^, plotted against Na^+^ cation fraction (*y*Na^+^). (a) Symbols: open circle, triangle, and square – results of this study for 1.0, 1.5, and 2.0 mol kg^–1^ ionic strength as indicated; open circle at *y*Na^+^ equal to 0 – measurement of Maksimov et al.;^7^ solid symbols at *y*Na^+^ equal to 0 – measurements of Macaskill and Bates^27^ (*I* = 1.0 mol kg^–1^) and Bates and Macaskill^19^ (*I* = 2.0 mol kg^–1^); solid symbols at *y*Na^+^ equal to 1.0 – from Harned’s rule coefficients in Table I of Harned.^24^ Lines: calculated using the Pitzer model, including values of ternary mixture parameters as described in the text. (b) The same data as in (a), but shown as the difference between measured and model-calculated activity coefficients (Δγ­(HCl)), with the measurements from this study adjusted by an equivalent of 0.5 mV (a factor of about 0.99 in γ_HCl_). Values of γ_HCl_ from other studies (at *y*Na^+^ equal to 0 and 1) are not adjusted. Symbols: circles, triangle, and square – all values for 1.0, 1.5, and 2.0 mol kg^–1^ ionic strength, respectively. The symbols for 1.0 mol kg^–1^ (circles), the measurements from which the adjustment was determined, are enlarged and shaded to contrast them with the other data. Error bars (Table S4) are included.

Figure 4a–c shows the approximately linear change of measured γ_HCl_ with temperature at all ionic strengths for the three values of *y*Na^+^. Figure 4d, for *y*Na^+^ equal to 0.5, presents values of γ_HCl_ at each temperature divided by the corresponding value at 40 °C. This normalization enables the relative slopes of γ_HCl_ with respect to temperature to be compared across all ionic strengths. The change in γ_HCl_ with temperature, for constant composition, is greatest at the highest ionic strength and appears to decrease smoothly: at ionic strength 5.5 mol kg^–1^ the value of γ_HCl_ at 5 °C is about 1.14 times that at 40 °C, whereas at 1.0 mol kg^–1^, the increase is only a factor of about 1.05.

**Figure 4:**
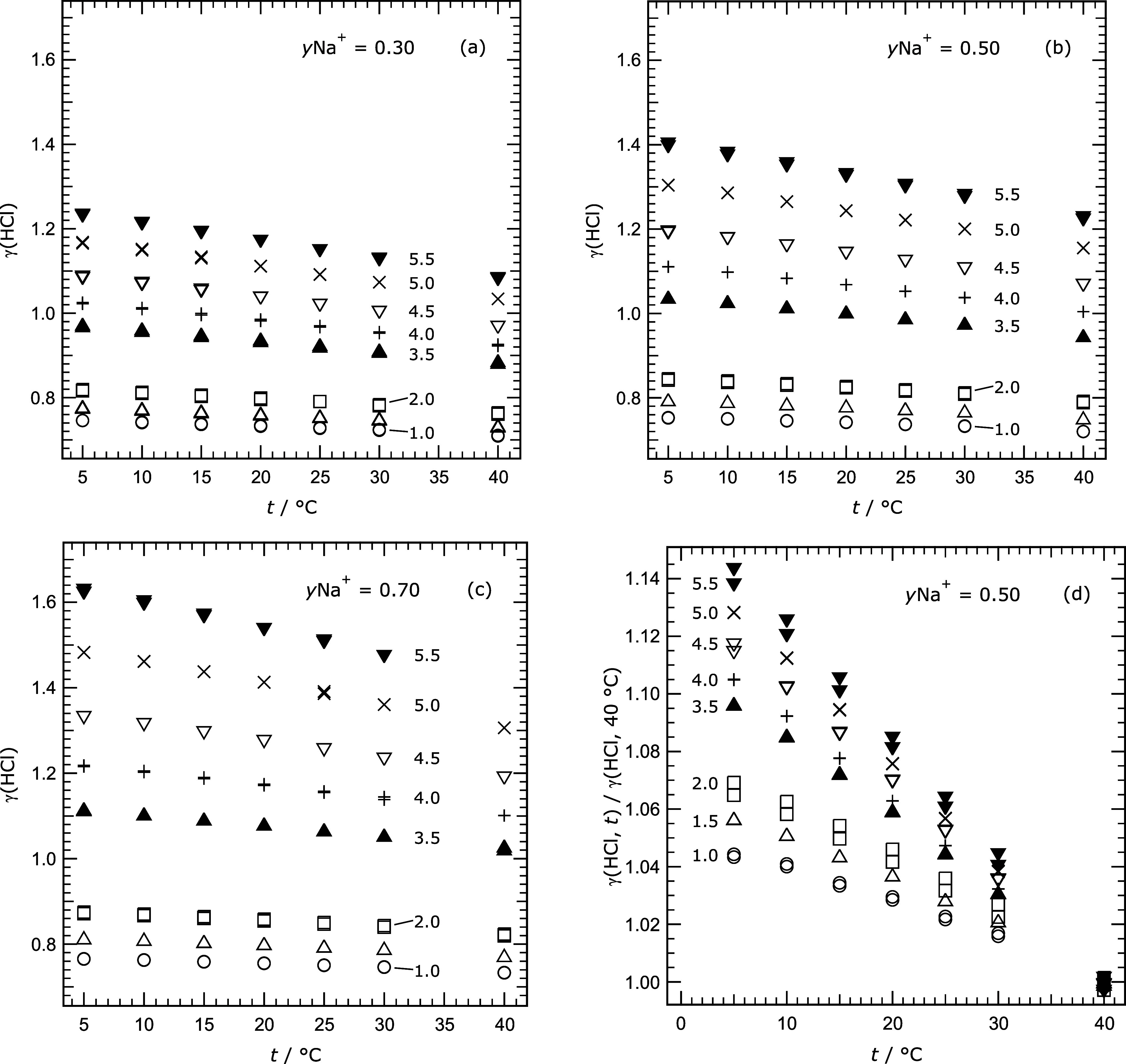
Measured mean activity coefficients γ_HCl_ (labeled γ­(HCl) for clarity) plotted against temperature (*t*) for different cation fractions *y*Na^+^ and ionic strengths as indicated on the plots. Symbols and ionic strengths: open circle – 1.0; triangle – 1.5; square and solid square – 2.0; solid triangle – 3.5; plus – 4.0; inverted triangle – 4.5; cross – 5.0; solid inverted triangle – 5.5 mol kg^–1^. Plots (a)–(c): for *y*Na^+^ = 0.30, 0.50, and 0.70, respectively. (d) The ratio of each γ_HCl_ to its value at 40 °C (for the same ionic strength and *y*Na^+^), to show how the variation of γ_HCl_ with temperature increases with ionic strength.

### 4.2 Tris Buffer (Equimolal TrisHCl and Tris) in Aqueous NaCl

For the aqueous HCl–NaCl–TrisHCl solutions discussed above, the molalities of both H^+^ and Cl^–^ are known and eq 1 was used to calculate the mean activity coefficient of HCl from the measured potentials. This is not possible for the solutions containing Tris buffer (0.04 mol kg^–1^ TrisHCl and Tris). In these alkaline solutions, the H^+^ content of the solutions is determined from the very slight dissociation of weak acid TrisH^+^ (to yield H^+^ and Tris), which is a function of the values of the thermodynamic equilibrium constant and activity coefficients of the three species. When considering the results of the measurements for these solutions, it is therefore helpful to define the following acidity function, *Q*, which can be calculated directly from the measured cell potentials:

5 $$Q=\ln\left(mH^{+}\cdot {\gamma_{\mathrm{HCl}}}^{2}\right)=\left(E^{0}-E\right)\cdot \left(F/\mathrm{RT}\right)-\ln\left(m{\mathrm{Cl}}^{-}\right)$$

Measured and adjusted cell potentials for these solutions are given in Table 5 together with values of the acidity function calculated from them. Table S8 of the Supporting Information contains the estimated uncertainties in the acidity function and information relevant to the adjustment of the measured potentials. Note that this function is a natural logarithm rather than decadal, and is without a reversal of sign so that all calculated values are negative.

**Table 5:** Harned Cell Results for 0.04 mol kg^–1^ Tris Buffer in Aqueous NaCl at Ionic Strengths of 0.2, 1.0, and 4.0 mol kg^–1^, Including Values of the Acidity Function (Equation 5)­^a^

cell	*t* (°C)	*m*Cl^–^ (mol kg^–1^)	*m*Tris (mol kg^–1^)	*m*TrisHCl (mol kg^–1^)	*m*NaCl (mol kg^–1^)	*E*(adj.) (V)	*u*(*E*)^b^ (mV)	acidity function *Q* ^c^	*u*(*Q*)
73	5	0.20	0.03998	0.04004	0.16000	0.76791	0.096	–20.6624	0.0042
74	5	0.20	0.04001	0.04001	0.16000	0.76778	0.096	–20.6567	0.0042
75	5	1.00	0.04001	0.04000	0.96004	0.73758	0.020	–21.0062	0.0014
76	5	1.00	0.04001	0.04001	0.96001	0.73757	0.020	–21.0060	0.0014
77	5	4.00	0.04001	0.03999	3.96013	0.70718	0.150	–21.1242	0.0064
78	5	4.00	0.03995	0.04003	3.96006	0.70697	0.150	–21.1155	0.0064
73	10	0.20	0.03998	0.04004	0.16000	0.76581	0.098	–20.2930	0.0042
74	10	0.20	0.04001	0.04001	0.16000	0.76568	0.098	–20.2872	0.0042
75	10	1.00	0.04001	0.04000	0.96004	0.73485	0.021	–20.6334	0.0014
76	10	1.00	0.04001	0.04001	0.96001	0.73485	0.021	–20.6331	0.0014
77	10	4.00	0.04001	0.03999	3.96013	0.70370	0.140	–20.7430	0.0059
78	10	4.00	0.03995	0.04003	3.96006	0.70351	0.140	–20.7351	0.0059
73	15	0.20	0.03998	0.04004	0.16000	0.76368	0.110	–19.9414	0.0046
74	15	0.20	0.04001	0.04001	0.16000	0.76353	0.110	–19.9352	0.0046
75	15	1.00	0.04001	0.04000	0.96004	0.73210	0.021	–20.2790	0.0014
76	15	1.00	0.04001	0.04001	0.96001	0.73209	0.021	–20.2786	0.0014
77	15	4.00	0.04001	0.03999	3.96013	0.70021	0.110	–20.3809	0.0046
78	15	4.00	0.03995	0.04003	3.96006	0.70006	0.110	–20.3748	0.0046
73	20	0.20	0.03998	0.04004	0.16000	0.76145	0.110	–19.6039	0.0045
74	20	0.20	0.04001	0.04001	0.16000	0.76130	0.110	–19.5979	0.0045
75	20	1.00	0.04001	0.04000	0.96004	0.72927	0.130	–19.9394	0.0053
76	20	1.00	0.04001	0.04001	0.96001	0.72909	0.130	–19.9320	0.0053
77	20	4.00	0.04001	0.03999	3.96013	0.69671	0.100	–20.0365	0.0042
78	20	4.00	0.03995	0.04003	3.96006	0.69657	0.100	–20.0310	0.0042
73	25	0.20	0.03998	0.04004	0.16000	0.75912	0.110	–19.2810	0.0045
74	25	0.20	0.04001	0.04001	0.16000	0.75897	0.110	–19.2750	0.0045
75	25	1.00	0.04001	0.04000	0.96004	0.72638	0.140	–19.6157	0.0056
76	25	1.00	0.04001	0.04001	0.96001	0.72618	0.140	–19.6081	0.0056
77	25	4.00	0.04001	0.03999	3.96013	0.69312	0.067	–19.7075	0.0029
78	25	4.00	0.03995	0.04003	3.96006	0.69311	0.067	–19.7071	0.0029
73	30	0.20	0.03998	0.04004	0.16000	0.75672	0.110	–18.9706	0.0044
74	30	0.20	0.04001	0.04001	0.16000	0.75656	0.110	–18.9646	0.0044
75	30	1.00	0.04001	0.04000	0.96004	0.72341	0.150	–19.3048	0.0059
76	30	1.00	0.04001	0.04001	0.96001	0.72319	0.150	–19.2966	0.0059
77	30	4.00	0.04001	0.03999	3.96013	0.68930	0.100	–19.3856	0.0040
78	30	4.00	0.03995	0.04003	3.96006	0.68944	0.100	–19.3910	0.0040
73	40	0.20	0.03998	0.04004	0.16000	0.75180	0.110	–18.3919	0.0046
74	40	0.20	0.04001	0.04001	0.16000	0.75165	0.110	–18.3861	0.0046
75	40	1.00	0.04001	0.04000	0.96004	0.71744	0.170	–18.7277	0.0066
76	40	1.00	0.04001	0.04001	0.96001	0.71720	0.170	–18.7188	0.0066
77	40	4.00	0.04001	0.03999	3.96013	0.68159	0.490	–18.7856	0.0183
78	40	4.00	0.03995	0.04003	3.96006	0.68228	0.490	–18.8111	0.0183

a Column *m*Cl^–^ contains rounded values, and exact molalities can be calculated from the listed *m*NaCl and *m*TrisHCl. Prefix “*u*“ in the column headers denotes an uncertainty. More complete results can be found in the Supporting Information.

b Cell potentials and their uncertainties are listed here to a fixed 5 digits and 3 digits following the decimal point, respectively. This was done for simplicity. In some cases the removal of a final zero in *u*(*E*) is preferred.^28^

c The acidity function *Q* is equal to ln­(*m*H^+^·γ_HCl_^2^), see eq 5. In the same way as for *E*(adj.) and *u*(*E*) we report both *Q* and *u*(*Q*) to a fixed 4 digits following the decimal point.

Figure 5a shows the acidity function (on a log_10_ basis) at all temperatures, together with values determined from measurements of DelValls and Dickson^1^ for artificial seawater containing the same stoichiometric molalities of TrisHCl and Tris as in our work. The top axis indicates the nominal practical salinities of artificial seawater corresponding to the ionic strengths on the bottom *x*-axis. The line on the plot represents values of the acidity function calculated for the Tris buffer in aqueous NaCl over a very wide range of ionic strength using the draft model of these solutions of Clegg et al.^6^ This includes parameters for the interaction of Tris with Na^+^ and a number of other ions, but not parameters for Na^+^–TrisH^+^–Cl^–^ or H^+^–TrisH^+^–Cl^–^ interactions (the latter do not influence the calculated acidity function for reasons explained in the following section).

**Figure 5:**
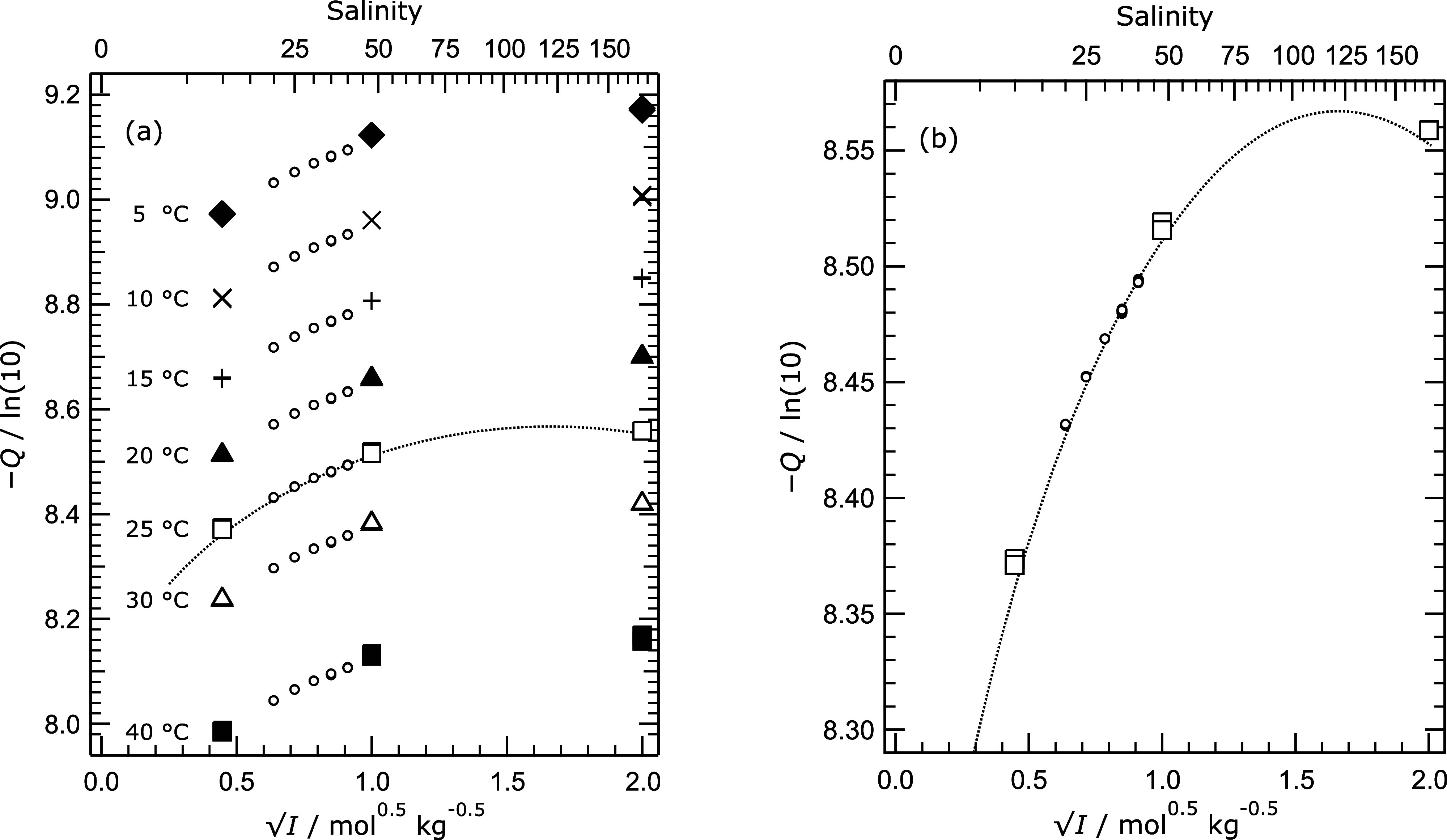
Values of the acidity function quantity −*Q*/ln­(10) (equivalent to −log_10_(*m*H^+^·γ_HCl_^2^), see eq 5), calculated from measured potentials and plotted against the square root of ionic strength *I* (mol kg^–1^). The upper *x*-axis is the salinity of an artificial seawater of the same ionic strength. (a) Symbols (data from this study): solid diamond – 5 °C; cross – 10 °C; plus – 15 °C; solid triangle – 20 °C; square – 25 °C; triangle – 30 °C; solid square – 40 °C. Symbols (data from DelValls and Dickson^1^ for Tris buffer in artificial seawater): small circles – values at all the indicated temperatures. Dotted line: values calculated using the model of Clegg et al.^6^ for 25 °C as described in the text. (a) For temperatures from 5 to 40 °C, as indicated. (b) Results for 25 °C only.

Figure 5b, which is for 25 °C only, demonstrates that our results for the Tris buffer in aqueous NaCl are quite close to those for an artificial seawater medium. The draft model yields values of the acidity function that are slightly lower than measured values, but the results are encouraging

### 4.3 Modeling

In this work, as in our previous study, we do not attempt to develop a Pitzer model of the solutions from the results. This is because such a model requires the consideration of a large range of literature information, and because further Harned cell data sets are still in preparation. Here we summarize the requirements for Pitzer models of the solutions studied in this work. In Table 6 we list the binary and ternary interactions for which the Pitzer model contains parameters.

**Table 6:** Binary and Ternary Interactions in the Pitzer Model for the Two Solutions Studied

(1) aqueous HCl–NaCl–TrisHCl		(2) Tris buffer in aqueous NaCl	
interactions	data available^a^	interactions^b^	data available^a^
H^+^–Cl^–^	yes	(H^+^–Cl^–^)	yes
Na^+^–Cl^–^	yes	Na^+^–Cl^–^	yes
TrisH^+^–Cl^–^	yes^c^	TrisH^+^–Cl^–^	yes^c^
H^+^–Na^+^–Cl^–^	yes	(H^+^–Na^+^–Cl^–^)	yes
H^+^–TrisH^+^–Cl^–^	yes^d^	(H^+^–TrisH^+^–Cl^–^)	yes^d^
Na^+^–TrisH^+^–Cl^–^	^e^	Na^+^–TrisH^+^–Cl^–^	^e^
		Tris–NaCl	yes^f^
		Tris–TrisHCl	yes^f^

a A “yes” in this column means that there are published data from which the Pitzer interaction parameters can be determined and/or values of the parameters available in the literature.

b The values in parentheses are needed for a calculation of speciation in the solution, but not for the cell potential (see eq 6).

c Currently available for 25 °C only.^19^

d See study of Maksimov et al.^7^

e Currently the subject of isopiestic measurements to determine osmotic coefficients (J. Miladinovic, pers. comm.), also studied by Tishchenko^20^ (see text).

f Studied by Lodeiro et al.^21^

For aqueous HCl–NaCl–TrisHCl, there are existing data from which most interactions can be quantified, although in two cases (TrisH^+^–Cl^–^ and Na^+^–TrisH^+^–Cl^–^) the information is mostly restricted to 25 °C. We note that Tishchenko^20^ has developed a Pitzer chemical speciation model of Tris buffer in aqueous NaCl, based largely on measurements made in the same study. Some of the results of Tishchenko have been examined by Lodeiro et al.,^21^ and seem likely to be erroneous—notably osmotic and activity coefficients of aqueous Tris solutions—or were found to be inconsistent with solubility measurements.

When considering a model for the Tris buffer in aqueous NaCl solutions and what can be determined from Harned cell measurements, it is helpful to substitute for *a*H^+^ in eq 1 as follows:

6 $$E=E^{0}-\mathrm{RT}/F\cdot \ln\left(K\left({\mathrm{TrisH}}^{+}\right)\cdot \left(m{\mathrm{TrisH}}^{+}\cdot m{\mathrm{Cl}}^{-}/m\mathrm{Tris}\right)\cdot {\gamma_{\mathrm{TrisHCl}}}^{2}/\gamma_{\mathrm{Tris}}\right)$$

and, substituting from eq 5

$$Q-\ln(K\left({\mathrm{TrisH}}^{+})\right)=\ln\left((m{\mathrm{TrisH}}^{+}/m\mathrm{Tris})\cdot {\gamma_{\mathrm{TrisHCl}}}^{2}/\gamma_{\mathrm{Tris}}\right)$$ 7

where *K*(TrisH^+^) (mol kg^–1^) is the acid dissociation constant of TrisH^+^. The value of this dissociation constant is known,^9^ and the molalities of TrisH^+^ and Tris at equilibrium will differ very little from their stoichiometric values (known from the preparation of the solutions). Consequently, the measured cell potentials can be used in eq 6 or eq 7 to obtain the quantity γ_TrisHCl_^2^/γ_Tris_. The list of Pitzer model interactions in Table 6 for the Tris buffer solutions is longer than that for aqueous HCl–NaCl–TrisHCl, but three of these interactions—the ones involving H^+^—do not influence the calculated cell potential (although they are required to calculate the H^+^ molality).

The availability of data that can be used to determine the value of the Pitzer model parameters for the various interactions in the buffer solutions is similar to that for aqueous HCl–NaCl–TrisHCl, as many interactions are common to both (Table 6). The additional ones, involving dissolved Tris, are either currently being measured (J. Miladinovic, pers. comm.), or have already been studied as a part of our project.^21^

The Harned cell measurements yield products and quotients of activity coefficients and, consequently, only sums and differences of a number of the Pitzer model interaction parameters can be determined from the data. We have listed these in Table 7 for both solutions.

**Table 7:** Pitzer Interaction Parameters That Occur as Sums and Differences in the Expressions for the Activity Products in the Equations for Cell Potentials

aqueous HCl–NaCl–TrisHCl		Tris buffer in aqueous NaCl	
interactions	parameters	interactions	parameters
Na^+^–Cl^–^, Na^+^–H^+^	β_Na,Cl_^(0)^ + θ_Na,H_ ^a^	Na^+^–Cl^–^, Na^+^–TrisH^+^, Tris–Na^+^	β_Na,Cl_^(0)^ + θ_Na,TrisH_ – λ_Tris,Na_ ^c^
Na^+^–Cl^–^, Na^+^–H^+^–Cl^–^	4*C*_Na,Cl_^(0)^ + ψ_H,Na,Cl_ ^a^	Na^+^–Cl^–^, Na^+^–TrisH^+^, Tris–Na^+^–Cl^–^	4*C*_Na,Cl_^(0)^ + ψ_Na,TrisH,Cl_ – ζ_Tris,Na,Cl_ ^c^
TrisH^+^–Cl^–^, TrisH^+^–H^+^	β_TrisH,Cl_^(0)^ + θ_TrisH,H_ ^b^	Na^+^–TrisH^+^–Cl^–^, Tris–Na^+^–Cl^–^	ψ_Na,TrisH,Cl_ + ζ_Tris,Na,Cl_ ^d^
TrisH^+^–Cl^–^, TrisH^+^–H^+^–Cl^–^	4*C*_TrisH,Cl_^(0)^ + ψ_H,TrisH,Cl_ ^b^	TrisH^+^–Cl^–^, Tris–TrisH^+^–Cl^–^	4*C*_TrisH,Cl_^(0)^ – ζ_Tris,TrisH,Cl_ ^e^

a Already known, and parameters are available in the literature.

b Can be determined from the study of Maksimov et al.,^7^ and other data for aqueous TrisHCl solutions.

c Parameters θ_Na,TrisH_ and ψ_Na,TrisH,Cl_ can be determined from osmotic coefficient measurements of NaCl–TrisHCl solutions currently underway (J. Miladinovic, pers. comm.), and λ_Tris,Na_ and ζ_Tris,Na,Cl_ from measurements of solubilities in aqueous NaCl–Tris solutions.^21^

d These parameters occur as a sum because *m*TrisH^+^ is equal to *m*Tris in the solutions.

e Parameters λ_Tris,TrisH_ and ζ_Tris,TrisH,Cl_ can be determined from measurements of solubilities in aqueous TrisHCl–Tris solutions.^21^

Typically, parameters for cation–anion interactions (e.g., Na^+^–Cl^–^, TrisH^+^–Cl^–^) will be known from other measurements, but this is not always true for other types of interaction. Consequently it is sometimes the case, for example in the analysis of various types of data by Lodeiro et al.^21^ for solutions containing Tris and/or TrisH^+^, that only the total value of a pair or set of three parameters can be determined. This must be taken into account in the development of models of the solutions.

## 5 Conclusions

We have measured cell potentials and obtained mean activity coefficients of HCl in aqueous HCl–NaCl–TrisHCl solutions at ionic strengths from 1.0 to 5.5 mol kg^–1^ and from 5 to 40 °C. Our two sets of measurements—for ionic strengths up to 2.0 mol kg^–1^, and for 3.5 mol kg^–1^ and above—were carried out several years apart, and the lower ionic strength measurements appear to have a small offset in the measured potentials. This deviation is likely related to the condition of the electrodes used for these particular solutions, and calculations presented here suggest that this can be corrected for. On the basis of comparisons made using data for 25 °C, our results are consistent with literature data for aqueous HCl–TrisHCl and HCl–NaCl solutions. The mean activity coefficients of HCl show smooth changes with temperature, as expected, and values are greatest at the highest ionic strengths and lowest temperatures.

We also measured cell potentials of a smaller number of aqueous solutions of NaCl containing equal stoichiometric molalities of Tris and TrisHCl, for the same range of temperatures and ionic strengths as those of aqueous HCl–NaCl–TrisHCl. The results, expressed in terms of an acidity function, are quite similar to those obtained for the same Tris buffer in artificial seawater by DelValls and Dickson^1^ and also agree satisfactorily with values predicted by the draft Pitzer model of Clegg et al.^6^ We have summarized interactions in the Pitzer model that apply to the solutions studied here, noting whether the corresponding parameters in the model (or data from which they can be determined) are available.

In combination with other literature data, including our previous study,^7^ these new measurements should enable a Pitzer ion-interaction model of the HCl–NaCl–TrisHCl solutions to be developed. This, together with the data for Tris buffer in aqueous NaCl, is an important step toward the development of a speciation model of acid–base equilibrium of Tris buffers in NaCl media and in artificial seawater solutions.

## Supplementary Material



## Notes

Description of electrode preparation; determination of standard potentials; densities, water activities, and *p*HCl of the measurement solutions; tabulation of original measured potentials; tabulation of derived γ_HCl_ and acidity function values including uncertainties (PDF)
