# Supplementary material for: Activity Coefficients of HCl in Solutions Related to “Tris” Buffers in Artificial Seawater. II. HCl + NaCl + TrisHCl + H2O, and Tris Buffer + NaCl + H2O, to High Ionic Strength and from 5 to 40 °C
Source: J Chem Eng Data. 2025 Aug 11;70(9):3614–29. doi: 10.1021/acs.jced.5c00369 (PMC12434662; doi:10.1021/acs.jced.5c00369)
Supplement: Supplementary file 1 [file je5c00369_si_001.pdf]

Supporting Information

Activity Coefficients of HCl in Solutions Related to 'Tris' Buffers in Artificial Seawater. II. HCl + NaCl + TrisHCl + H_2O, and Tris buffer + NaCl + H_2O, to High Ionic Strength and From 5 °C to 40 °C

Igor Maksimov,[a*] Toshiaki Asakai,[a] Yuya Hibino,[a] and Simon L. Clegg[b*]

[a] National Metrology Institute of Japan, National Institute of Advanced Industrial Science and Technology (AIST), 1-1-1 Umezono, Tsukuba, Ibaraki 305-8563, Japan
[b] School of Environmental Sciences, University of East Anglia, Norwich NR4 7TJ, United Kingdom

* Corresponding authors. *E-mail*: maksimov.igor@aist.go.jp, and s.clegg@uea.ac.uk

Contents

	Page
1. Use of the Harned cells in this study	2
2. Determination of standard potentials, E^0	3
3. Densities of the solutions	4
4. Estimation of the water activities and *p*HCl of the solutions	5
5. Tabulation of the results	6
6. Tables	7
References	21

This Supporting Information (SI) describes, first of all, some of the particular difficulties experienced in the Harned cell measurements in this study. Next there is further information, supporting the description in the main text, on the determination of standard potentials of the cells (E^0). We also describe the estimation of the densities of the solutions, their water activities, and equilibrium partial pressures of HCl which are needed in order to adjust cell potentials to a standard 1 atm pressure of H_2.

The complete experimental results are tabulated, including the measured potentials, ambient pressures, densities and water activities of the solutions, and the mean activity coefficients of HCl and acidity functions and their estimated uncertainties.

1. Use of the Harned cells in this study

An NMIJ Harned Cell is shown in Figure S1, below. This is the same image as in the Supporting Information to Maksimov et al.[1] which describes the preparation of the electrodes and operation of the cell in detail. That work also includes comments from Andrew G. Dickson of the Scripps Institution of Oceanography concerning the preparation of electrodes in his laboratory, where different.

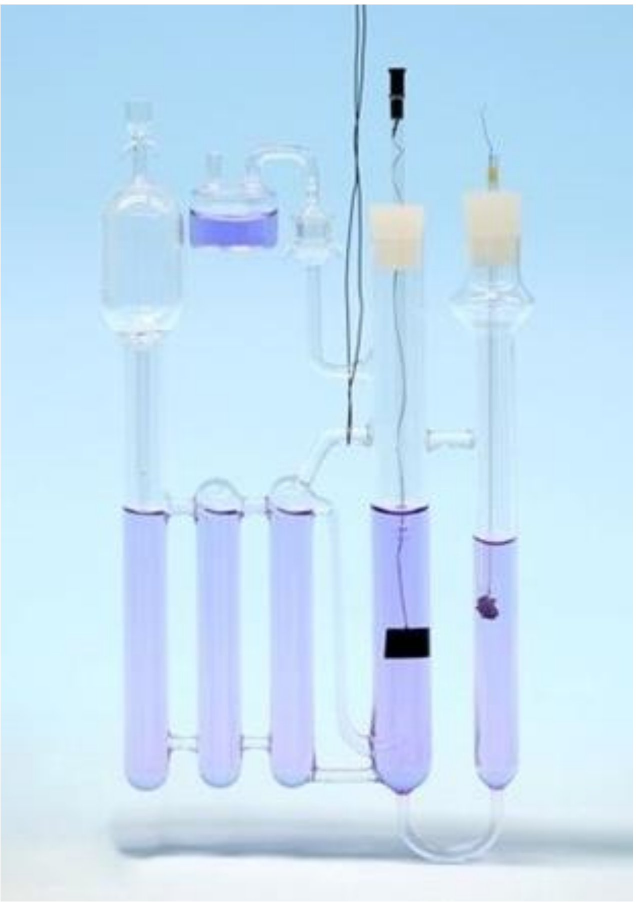

Figure S1. An NMIJ Harned cell. The platinum hydrogen electrode is the dark square in the left half of the U-shaped compartment, and the irregular round shape in the right half is the silver – silver chloride electrode.

The following features of the measurements made in this study may affect the accuracy of the results.

(i) In the most concentrated solutions (those containing 4.0 mol kg^{-1} Cl^- or higher) some salt crystallisation was observed around the input capillary tube in the first pre-saturator tube near the end of the two day measurement period, i.e. 40-50 hours after the start of hydrogen bubbling. This interrupted the hydrogen flow by clogging the capillary tube. An example of the crystallisation is shown in Figure S2. It seems reasonable to attribute the observed phenomenon to the solubilities of TrisHCl or NaCl being exceeded, due to the loss of some water to the passing H_2 flow which is initially dry as it enters the first pre-saturator. The loss of water vapor is a continuous process in all the measurement cycles, but is greatest at the highest temperatures of 35 and 40 ºC due to the increased water partial pressure (about a factor of 8.5 greater at 40 °C compared to 5 ºC). At the end of the two day measurement cycle the loss of water from the solution in the compartment of the first pre-saturator was clearly visible. The vulnerability of the NMIJ Harned cell to the effects of water evaporation we attribute to its relatively small volume, enough to contain about 80 mL of tested solution, in combination with the H_2 gas flow rate. A total of eleven measured potentials were discarded because of these difficulties.

The chemical compositions of studied solutions in the electrode compartments are thought to have remained stable – in the cases where no salt crystallisation was observed – because of the use of the three pre-saturators in series, but a greater drift in the measured potentials with time was observed for some of the most concentrated solutions at the highest temperatures as noted in section 2.2 in the main text of this work.

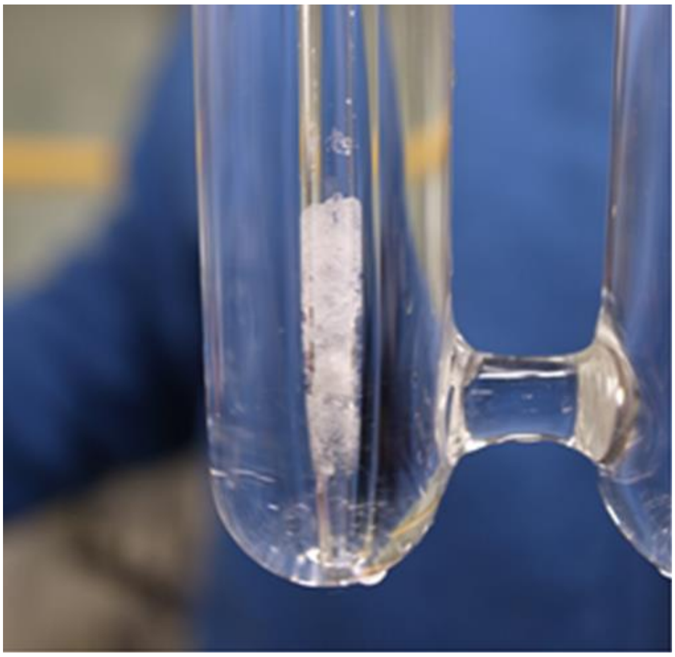

Figure S2. Bottom of the first pre-saturator cell, after the end of a measurement cycle, showing the input capillary tube with salt crystallisation near the tip.

(ii) Loss of platinum black coating of the H_2 electrode near end of a measurement cycle was observed in cells 95 and 107, for reasons that are not yet understood. The phenomenon causes very large changes in cell potential (compared to the cells containing duplicates of the same solutions) and the results were therefore discarded. (We note that the coating of the platinum H_2 electrodes is routinely renewed for every measurement cycle.)

(iii) The set of reference electrodes used for the measurements of the 1.0, 1.5, and 2.0 mol kg^{-1} Cl^- solutions (all in 2023) was different from that used in 2017. The low Cl^- molality measurements were the final use of these $Ag_{(s)}$/$AgCl_{(s)}$ electrodes, which had a relatively small size of original silver bulb, ca. 80-100 mg instead of the standard mass of 200 mg. During the measurements we observed some discoloration of the bulb, mostly likely caused by depletion of the $AgCl_{(s)}$ layer in the solutions containing high molalities of Cl^- (the solubility of AgCl is known to *increase* with Cl^- molality, see Maksimov et al.[1]). The typical work of NMIJ, and other national metrological institutes that use Harned cells, is for the measurement of dilute solutions in which electrode degradation or dissolution does not occur. Thus we were unaware of the problem initially. We have nonetheless retained the results for the low molality Cl^- solutions (in Table 4 in the main text and Table S7 here), despite the fact that they appear to show some offsets relative to the other measurements.

2. Determination of standard potentials, E^0

The cells used for measurements of the 0.01 mol kg^{-1} HCl to determine E^0 at each temperature are listed in Table S1, and measured potentials (adjusted to exactly 0.01 m HCl) in Table S2. The results for the measurements made in 2017 are the same as those listed by Maksimov et al.[1] and described in

the Supporting Information to that work. We used the same approach, based upon voltage drift, to determine uncertainties of the standard potentials obtained from measurements of 0.01 mol kg^{-1} HCl in 2023. The values were found to be the same as for the 2017 data, with the exception of the value at 40 °C (0.000049 mV compared to 0.000057 mV). We also note that the results of measurements of the standard potential of cells G1-L1 and M1-R1 in 2022-2023 agree well within determined uncertainty limits, except at the highest temperature (probably due to the varying impact of evaporation), even though these measurements are separated by a half-year.

The uncertainties in E^0 listed in Table S2 were calculated using standard methods of uncertainty propagation (e.g., JCGM[2]). The standard uncertainties in the temperature, HCl molality and atmospheric pressure were 0.01 K (thermometer certificate), 5×10^{-6} mol kg^{-1} (coulometric titration), and 20 Pa (barometer certificate), respectively.

3. Densities of the solutions

The densities of the solutions, at each experimental temperature, are used in the final term in eq 2 for the adjustment of the measured potentials to pH_2 equal to 1 atm. The effect on the potential of this final term, "JetCorr", is about 0.02 mV (Maksimov et al.[1]). However, when the HCl mean activity is being calculated the overall influence on γ_{HCl} is that of the difference between the JetCorr terms for the test solution and for the 0.01 *m* HCl used to obtain E^0. This will be a difference of the order of only a few μV.

Densities of the solutions were estimated by assuming additivity of apparent molar volumes of the solutes HCl, NaCl, TrisHCl, and Tris in the solution:

$$V_{\mathrm{Tot.}} = m\mathrm{HCl}\cdot V^{\Phi}(\mathrm{HCl}) + m\mathrm{NaCl}\cdot V^{\Phi}(\mathrm{NaCl}) + m\mathrm{TrisHCl}\cdot V^{\Phi}(\mathrm{TrisHCl}) + m\mathrm{Tris}\cdot V^{\Phi}(\mathrm{Tris}) + 1000/\rho(\mathrm{Water}) \quad \text{(S1)}$$

where $V_{\mathrm{Tot.}}$ is the estimated total volume of a solution containing 1000 g H_2O, V^{Φ} (cm^3 mol^{-1}) is the apparent molar volume of the indicated solute at its molality (*m*) in the mixture, and ρ(Water) is the density of pure water in g cm^{-3}. The density of the solution mixture is obtained from the total volume as given above, and the total mass (based upon the same 1000 g of water solvent).

Densities of pure water, and apparent molar volumes of aqueous HCl and aqueous TrisHCl, were obtained as described in the Supporting Information to Maksimov et al.[1] Values for aqueous NaCl were obtained from Clegg and Wexler,[3] and Tris from the following equation fitted to the data of Ford et al.:[4]

$$V^{\Phi}(\mathrm{Tris}) = 69.26 + 0.0754\cdot T \quad \text{(S2)}$$

Uncertainties in the densities of the solutions make a small contribution to those of the adjustments of the measured potentials to a pH_2 of 1 atm. For the HCl-NaCl-TrisHCl solutions we here assume that the uncertainties in the estimated densities are the same, at the same Cl^- molalities, as those estimated for aqueous HCl-TrisHCl solutions by Maksimov et al.[1] We have interpolated or extrapolated to obtain estimates at Cl^- molalities that occur only in this work and not that of Maksimov et al. Values of the uncertainties for these solutions are listed in Table S3. The solutions containing Tris buffer in aqueous NaCl are either dominated by NaCl (the solutions at 1.0 and 4.0

mol kg-1 ionic strength), or are so dilute that any errors in the estimated densities will be very small. Consequently, for these solutions we assume that the errors in the estimated densities are zero.

4. Estimation of the water activities and *p*HCl of the solutions

The approach used in this work is very similar to that for the HCl-TrisHCl solutions described in the Supporting Information to Maksimov et al.[1] The water activities of the solutions, at each experimental temperature, are used in the pH_2O term in eq 2 for the adjustment of cell potentials to exactly 1 atm pH_2. These water activities are calculated using the Pitzer model. For the HCl-NaCl-TrisHCl solutions we use $TrisH^+$-Cl^- parameters from Table 2 of Tishchenko,[5] those for H^+-Cl^- from Holmes et al.,[6] and those for Na^+-Cl^- from Archer.[7] We also use mixture parameters for H^+-$TrisH^+$-Cl^- interactions (known at 25 °C only) from Bates and Macaskill,[8] and those for H^+-Na^+-Cl^- (also known at 25 °C only) from Harvie and Weare.[9] Calculated water activities at 5, 25, and 40 °C for representative solutions are listed in Table S4. The contributions of the pH_2O term to the adjusted values of the cell potentials are largest at the highest measured temperatures, as is illustrated for HCl-TrisHCl solutions in section 4 of the Supporting Information to Maksimov et al.[1] As can be seen in Table S4, the water activities of the HCl-NaCl-TrisHCl solutions vary little with temperature, and water activities calculated for 25 °C (for which Pitzer parameters are generally more accurately known) could have been used at all temperatures without much loss of accuracy.

For the solutions of Tris buffer in aqueous NaCl the same Pitzer parameters as noted above were used, plus that for Tris-Na^+ interactions ($\lambda_{Tris,Na}$, invariant with temperature) from Millero et al.,[10] which is similar in value to the estimates from other data discussed by Lodeiro et al.[11]

Estimates of uncertainties of the water activities for the HCl-NaCl-TrisHCl solutions were made in the following way. First, we repeated the calculation of water activities but with the mixture parameters $\theta_{Na,TrisH}$ (-0.0045) and $\psi_{Na,TrisH,Cl}$ (-0.00283). These values were determined for $\theta_{Na,NH4}$ and $\psi_{Na,NH4,Cl}$ by Ji et al.,[12] and have been adopted as estimates. Second, for each total chloride molality and temperature, we determined the mean difference between the two sets of calculated water activities. Where the absolute values of these differences were below 0.00005 we assumed an uncertainty equal to ±0.00005, and where they were greater we assumed values equal to 0.00005 plus 75% of the difference between the two. While somewhat arbitrary, this procedure accounts for the fact that calculated water activities at higher ionic strengths will have greater uncertainties. The smaller influence of temperature will be less well accounted for. The estimated uncertainties in the water activities of the solutions are listed in Table S5.

The uncertainties of the water activities of the Tris buffer in aqueous NaCl solutions are neglected because the solutions either consist mainly of aqueous NaCl (at the two higher ionic strengths), for which the water activities are accurately known, or very dilute for which there is a low sensitivity of water activity to solution composition.

In the same way as described by Maksimov et al.,[1] equilibrium partial pressures of HCl above the HCl-NaCl-TrisHCl measurement solutions were calculated using the expression:

$$pHCl = aH^+ \cdot aCl^- / K_H \qquad (S3)$$

where K_H ($mol^2\ kg^{-2}\ atm^{-1}$) is the Henry's law constant of HCl at the temperature of interest, given by:

$$\ln(K_H) = 4.6187 + 5977.5014/T - 0.03401 \cdot T \quad (S4)$$

The highest pHCl occurs for the 5 mol kg^{-1} chloride solution for which yNa^+ is equal to 0.5, and is about 0.19×10^{-5} atm at 40 °C, which corresponds to a negligible change in the adjustment of a measured potential. Therefore we did not estimate uncertainties of the partial pressures. The Tris buffer in aqueous NaCl solutions are alkaline, with a negligible equilibrium pHCl which is therefore neglected.

5. Tabulation of the results

Complete experimental results are listed in Tables S6 to S8. These contain the pressure, P, for each measurement; cell potentials including the original measured values (before the correction to pH_2 equal to 1 atm); the estimated uncertainties of the HCl mean activity coefficient or acidity function obtained from the measurements; and the water activity and density of each solution (used in the pressure correction).

6. Tables

Table S1. Cells used to measure 0.01 *m* HCl and determine standard potentials E^0 at different temperatures [a]

t / °C	Cells [b]	Cells [c]		*t* / °C	Cells [b]	Cells [c]
5	A–F, M–R, S–X	G1-L1, M1-R1		30	G–L, M–R, S–X	G1-L1, M1-R1
10	G–L, M–R, S–X	G1-L1, M1-R1		35	A1–F1	
15	A–F, M–R, S–X, A1–F1	G1-L1, M1-R1		40	G–L, M–R, S–X	M1-R1
20	A–F, M–R, S–X	G1-L1, M1-R1		45	A1–F1	
25	A–F, M–R, S–X	G1-L1, M1-R1				

[a] See Table 2 in the main text for the dates of the measurements.
[b] Measurements carried out in 2017.
[c] Measurements from 2022 and 2023.

Table S2. Standard potentials (E^0) and their uncertainties (u) determined in this work

t (°C)	E^0 (V) [a]	u(E^0) (V)		*t* (°C)	E^0 (V) [b]	u(E^0) (V)
5	0.234076	0.000027		5	0.234175	0.000027
10	0.231422	0.000028		10	0.231536	0.000028
15	0.228573	0.000028		15	0.228700	0.000028
20	0.225610	0.000033		20	0.225762	0.000033
25	0.222463	0.000034		25	0.222611	0.000034
30	0.219192	0.000034		30	0.219397	0.000034
35	0.215696	0.000045		-		
40	0.212104	0.000057		40	0.212064	0.000049
45	0.208306	0.000060		-		

[a] Measurements carried out in 2017.
[b] Measurements from 2022 and 2023.

Table S3. Estimated uncertainties (u) in calculated densities (ρ) of the HCl-NaCl-TrisHCl solution mixtures at different chloride molalities [a]

mCl$^-$ (mol kg^{-1})	u(ρ) (g cm^{-3})	mCl$^-$ (mol kg^{-1})	u(ρ) (g cm^{-3})
1.0	0.00079	4.0	-0.0012
1.5	0.00079	4.5	-0.0016
2.0	0.00106	5.0	-0.0019
3.5	0.0011	5.5	-0.0022

[a] The uncertainties are assumed to be the same at all temperatures.

Table S4. Calculated water activities of some of the HCl-NaCl-TrisHCl and Tris buffer in aqueous NaCl solutions at three temperatures [a]

mCl$^-$ (mol kg^{-1})	aH$_2$O (5 °C)	aH$_2$O (25 °C)	aH$_2$O (40 °C)
1.0	0.9674	0.9672	0.9672
2.0	0.9346	0.9337	0.9335
3.5	0.8829	0.8809	0.8804
4.5	0.8469	0.8442	0.8437
5.5	0.8097	0.8067	0.8064
0.2 (buffer) [b]	0.9927	0.9927	0.9927
1.0 (buffer) [b]	0.9666	0.9961	0.9660
4.0 (buffer) [b]	0.8540	0.8507	0.8497

[a] The value of yNa$^+$ is equal to 0.5 (HCl-NaCl-TrisHCl solutions).
[b] Tris buffer in aqueous NaCl.

Table S5. Estimated uncertainties in the water activities of the HCl-NaCl-TrisHCl solutions at several total Cl$^-$ molalities and all temperatures [a]

	Estimated uncertainty in water activity at the indicated Cl$^-$ molality				
t (°C)	1.0 mol kg^{-1}	2.0 mol kg^{-1}	3.5 mol kg^{-1}	4.5 mol kg^{-1}	5.5 mol kg^{-1}
5	0.000050	0.00022	0.00090	0.0017	0.0029
10	0.000050	0.00022	0.00089	0.0017	0.0029
15	0.000050	0.00022	0.00090	0.0017	0.0029
20	0.000050	0.00022	0.00090	0.0017	0.0029
25	0.000050	0.00022	0.00090	0.0017	0.0029
30	0.000050	0.00022	0.00090	0.0017	0.0029
35	0.000050				
40	0.000050	0.00022	0.00090	0.0018	0.0030

[a] There are no measurements of potential for the temperatures and chloride molalities for which the entries are blank.

Table S6. Harned cell results for HCl-NaCl-TrisHCl solutions for ionic strengths of 3.5 mol kg^{-1} and above [a]

Cell	*t* (°C) [b]	*P* (atm)	*m*Cl$^-$ (mol kg^{-1})	*y*Na$^+$	*m*HCl (mol kg^{-1})	*m*TrisHCl (mol kg^{-1})	*m*NaCl (mol kg^{-1})	*E*(meas.) (V) [c]	*E* (V) [c]	*E*(adj.) (V) [c]	u(*E*) (mV)	γ_{HCl}	u(γ_{HCl})	*a*H_2O	ρ (g cm^{-3})
79	5	0.996250	3.5	0.30	0.10012	2.37995	1.02003	0.260769	0.260890	0.260894	0.130	0.9656	0.00268	0.8866	1.1303
80	5	0.996250	3.5	0.30	0.10011	2.37985	1.02004	0.260587	0.260708	0.260712	0.130	0.9693	0.00269	0.8866	1.1303
81	5	0.996250	3.5	0.50	0.10005	1.70001	1.70007	0.257492	0.257613	0.257616	0.011	1.0342	0.00063	0.8829	1.1313
82	5	0.996250	3.5	0.50	0.10007	1.69994	1.70007	0.257496	0.257617	0.257620	0.011	1.0341	0.00063	0.8829	1.1313
83	5	0.996250	3.5	0.70	0.10002	1.01997	2.38012	0.254108	0.254229	0.254232	0.075	1.1101	0.00185	0.8793	1.1313
84	5	0.996250	3.5	0.70	0.10007	1.01992	2.38007	0.254003	0.254124	0.254127	0.075	1.1122	0.00185	0.8793	1.1313
79	10	0.993180	3.5	0.30	0.10012	2.37995	1.02003	0.259071	0.259271	0.259249	0.140	0.9547	0.00280	0.8861	1.1281
80	10	0.993180	3.5	0.30	0.10011	2.37985	1.02004	0.258874	0.259074	0.259052	0.140	0.9586	0.00281	0.8861	1.1281
81	10	0.993180	3.5	0.50	0.10005	1.70001	1.70007	0.255674	0.255873	0.255851	0.011	1.0239	0.00063	0.8823	1.1291
82	10	0.993180	3.5	0.50	0.10007	1.69994	1.70007	0.255680	0.255879	0.255857	0.011	1.0236	0.00063	0.8823	1.1291
83	10	0.993180	3.5	0.70	0.10002	1.01997	2.38012	0.252165	0.252364	0.252342	0.012	1.1004	0.00069	0.8786	1.1291
84	10	0.993180	3.5	0.70	0.10007	1.01992	2.38007	0.252157	0.252356	0.252334	0.012	1.1003	0.00069	0.8786	1.1291
79	15	0.993062	3.5	0.30	0.10012	2.37995	1.02003	0.257304	0.257562	0.257548	0.140	0.9423	0.00271	0.8856	1.1258
80	15	0.993062	3.5	0.30	0.10011	2.37985	1.02004	0.257104	0.257362	0.257348	0.140	0.9462	0.00272	0.8856	1.1258
81	15	0.993062	3.5	0.50	0.10005	1.70001	1.70007	0.253797	0.254054	0.254041	0.011	1.0116	0.00061	0.8818	1.1268
82	15	0.993062	3.5	0.50	0.10007	1.69994	1.70007	0.253804	0.254061	0.254048	0.011	1.0114	0.00061	0.8818	1.1268
83	15	0.993062	3.5	0.70	0.10002	1.01997	2.38012	0.250176	0.250432	0.250419	0.011	1.0883	0.00066	0.8779	1.1268
84	15	0.993062	3.5	0.70	0.10007	1.01992	2.38007	0.250169	0.250425	0.250412	0.011	1.0882	0.00066	0.8779	1.1268
79	20	0.995065	3.5	0.30	0.10012	2.37995	1.02003	0.255426	0.255734	0.255694	0.140	0.9306	0.00265	0.8852	1.1234
80	20	0.995065	3.5	0.30	0.10011	2.37985	1.02004	0.255222	0.255530	0.255490	0.140	0.9344	0.00266	0.8852	1.1234
81	20	0.995065	3.5	0.50	0.10005	1.70001	1.70007	0.251844	0.252150	0.252111	0.011	0.9993	0.00069	0.8813	1.1244
82	20	0.995065	3.5	0.50	0.10007	1.69994	1.70007	0.251851	0.252157	0.252118	0.011	0.9991	0.00069	0.8813	1.1244
83	20	0.995065	3.5	0.70	0.10002	1.01997	2.38012	0.248088	0.248393	0.248353	0.011	1.0766	0.00074	0.8774	1.1245
84	20	0.995065	3.5	0.70	0.10007	1.01992	2.38007	0.248085	0.248390	0.248350	0.011	1.0764	0.00074	0.8774	1.1245
79	25	0.993960	3.5	0.30	0.10012	2.37995	1.02003	0.253412	0.253835	0.253772	0.150	0.9174	0.00275	0.8849	1.1209
80	25	0.993960	3.5	0.30	0.10011	2.37985	1.02004	0.253203	0.253626	0.253563	0.150	0.9212	0.00276	0.8849	1.1209
81	25	0.993960	3.5	0.50	0.10005	1.70001	1.70007	0.249748	0.250170	0.250106	0.021	0.9856	0.00077	0.8809	1.1220
82	25	0.993960	3.5	0.50	0.10007	1.69994	1.70007	0.249755	0.250177	0.250113	0.021	0.9853	0.00077	0.8810	1.1220
83	25	0.993960	3.5	0.70	0.10002	1.01997	2.38012	0.245856	0.246276	0.246213	0.020	1.0633	0.00082	0.8770	1.1221
84	25	0.993960	3.5	0.70	0.10007	1.01992	2.38007	0.245857	0.246277	0.246214	0.020	1.0630	0.00082	0.8770	1.1221

79	30	0.992154	3.5	0.30	0.10012	2.37995	1.02003	0.251208	0.251791	0.251698	0.140	0.9052	0.00250	0.8847	1.1184
80	30	0.992154	3.5	0.30	0.10011	2.37985	1.02004	0.251019	0.251602	0.251509	0.140	0.9085	0.00251	0.8847	1.1184
81	30	0.992154	3.5	0.50	0.10005	1.70001	1.70007	0.247481	0.248061	0.247969	0.050	0.9725	0.00113	0.8807	1.1195
82	30	0.992154	3.5	0.50	0.10007	1.69994	1.70007	0.247487	0.248067	0.247975	0.050	0.9723	0.00113	0.8807	1.1195
83	30	0.992154	3.5	0.70	0.10002	1.01997	2.38012	0.243448	0.244026	0.243934	0.050	1.0507	0.00122	0.8767	1.1197
84	30	0.992154	3.5	0.70	0.10007	1.01992	2.38007	0.243455	0.244033	0.243941	0.050	1.0503	0.00122	0.8767	1.1197
79	40	0.992272	3.5	0.30	0.10012	2.37995	1.02003	0.246370	0.247362	0.247328	0.200	0.8790	0.00339	0.8845	1.1132
80	40	0.992272	3.5	0.30	0.10011	2.37985	1.02004	0.246115	0.247107	0.247073	0.200	0.8832	0.00341	0.8845	1.1132
81	40	0.992272	3.5	0.50	0.10005	1.70001	1.70007	0.242587	0.243575	0.243541	0.090	0.9432	0.00186	0.8804	1.1144
82	40	0.992272	3.5	0.50	0.10007	1.69994	1.70007	0.242589	0.243577	0.243543	0.090	0.9431	0.00186	0.8804	1.1144
83	40	0.992272	3.5	0.70	0.10002	1.01997	2.38012	0.238107	0.239091	0.239056	0.250	1.0251	0.00488	0.8763	1.1147
84	40	0.992272	3.5	0.70	0.10007	1.01992	2.38007	0.238441	0.239425	0.239390	0.250	1.0185	0.00485	0.8763	1.1147
85	5	1.006622	4.0	0.30	0.10022	2.72986	1.17003	0.254937	0.254931	0.254935	0.088	1.0222	0.00196	0.8700	1.1441
86	5	1.006622	4.0	0.30	0.10021	2.72974	1.16997	0.254813	0.254808	0.254811	0.088	1.0250	0.00197	0.8700	1.1441
87	5	1.006622	4.0	0.50	0.10016	1.94991	1.95005	0.250979	0.250973	0.250977	0.012	1.1106	0.00068	0.8651	1.1459
88	5	1.006622	4.0	0.50	0.10016	1.94975	1.94994	0.250988	0.250982	0.250986	0.012	1.1104	0.00068	0.8651	1.1459
89	5	1.006622	4.0	0.70	0.10006	1.16994	2.73009	0.246569	0.246562	0.246566	0.074	1.2182	0.00200	0.8601	1.1466
90	5	1.006622	4.0	0.70	0.10008	1.16994	2.73009	0.246673	0.246666	0.246670	0.074	1.2154	0.00200	0.8601	1.1466
85	10	1.006277	4.0	0.30	0.10022	2.72986	1.17003	0.253217	0.253253	0.253231	0.099	1.0097	0.00213	0.8694	1.1417
86	10	1.006277	4.0	0.30	0.10021	2.72974	1.16997	0.253078	0.253114	0.253092	0.099	1.0127	0.00214	0.8695	1.1417
87	10	1.006277	4.0	0.50	0.10016	1.94991	1.95005	0.249151	0.249186	0.249164	0.012	1.0978	0.00069	0.8644	1.1435
88	10	1.006277	4.0	0.50	0.10016	1.94975	1.94994	0.249159	0.249194	0.249172	0.012	1.0977	0.00069	0.8644	1.1435
89	10	1.006277	4.0	0.70	0.10006	1.16994	2.73009	0.244623	0.244657	0.244635	0.076	1.2052	0.00200	0.8593	1.1442
90	10	1.006277	4.0	0.70	0.10008	1.16994	2.73009	0.244729	0.244763	0.244741	0.076	1.2024	0.00200	0.8593	1.1442
85	15	1.008369	4.0	0.30	0.10022	2.72986	1.17003	0.251432	0.251493	0.251480	0.130	0.9955	0.00267	0.8688	1.1392
86	15	1.008369	4.0	0.30	0.10021	2.72974	1.16997	0.251245	0.251306	0.251293	0.130	0.9993	0.00268	0.8688	1.1392
87	15	1.008369	4.0	0.50	0.10016	1.94991	1.95005	0.247260	0.247320	0.247307	0.012	1.0831	0.00066	0.8637	1.1410
88	15	1.008369	4.0	0.50	0.10016	1.94975	1.94994	0.247268	0.247328	0.247315	0.012	1.0830	0.00066	0.8637	1.1410
89	15	1.008369	4.0	0.70	0.10006	1.16994	2.73009	0.242605	0.242664	0.242651	0.086	1.1902	0.00217	0.8586	1.1417
90	15	1.008369	4.0	0.70	0.10008	1.16994	2.73009	0.242725	0.242784	0.242771	0.086	1.1872	0.00216	0.8586	1.1417
85	20	1.007086	4.0	0.30	0.10022	2.72986	1.17003	0.249488	0.249636	0.249596	0.130	0.9817	0.00261	0.8683	1.1366
86	20	1.007086	4.0	0.30	0.10021	2.72974	1.16997	0.249301	0.249449	0.249409	0.130	0.9854	0.00262	0.8683	1.1366
87	20	1.007086	4.0	0.50	0.10016	1.94991	1.95005	0.245239	0.245385	0.245345	0.011	1.0681	0.00074	0.8631	1.1385
88	20	1.007086	4.0	0.50	0.10016	1.94975	1.94994	0.245244	0.245390	0.245350	0.011	1.0681	0.00074	0.8631	1.1384
89	20	1.007086	4.0	0.70	0.10006	1.16994	2.73009	0.240481	0.240626	0.240586	0.085	1.1742	0.00212	0.8580	1.1393

90	20	1.007086	4.0	0.70	0.10008	1.16994	2.73009	0.240600	0.240745	0.240705	0.085	1.1714	0.00212	0.8580	1.1393
85	25	1.005270	4.0	0.30	0.10022	2.72986	1.17003	0.247419	0.247686	0.247623	0.120	0.9667	0.00235	0.8680	1.1340
86	25	1.005270	4.0	0.30	0.10021	2.72974	1.16997	0.247246	0.247513	0.247450	0.120	0.9701	0.00236	0.8680	1.1340
87	25	1.005270	4.0	0.50	0.10016	1.94991	1.95005	0.243067	0.243332	0.243268	0.020	1.0525	0.00081	0.8627	1.1359
88	25	1.005270	4.0	0.50	0.10016	1.94975	1.94994	0.243072	0.243337	0.243273	0.020	1.0525	0.00081	0.8627	1.1359
89	25	1.005270	4.0	0.70	0.10006	1.16994	2.73009	0.238207	0.238470	0.238406	0.100	1.1576	0.00238	0.8575	1.1368
90	25	1.005270	4.0	0.70	0.10008	1.16994	2.73009	0.238345	0.238608	0.238544	0.100	1.1544	0.00238	0.8575	1.1368
85	30	1.002250	4.0	0.30	0.10022	2.72986	1.17003	0.245166	0.245602	0.245509	0.120	0.9527	0.00228	0.8677	1.1313
86	30	1.002250	4.0	0.30	0.10021	2.72974	1.16997	0.245019	0.245455	0.245362	0.120	0.9555	0.00228	0.8677	1.1313
87	30	1.002250	4.0	0.50	0.10016	1.94991	1.95005	0.240733	0.241166	0.241073	0.050	1.0374	0.00120	0.8624	1.1332
88	30	1.002250	4.0	0.50	0.10016	1.94975	1.94994	0.240738	0.241171	0.241078	0.050	1.0374	0.00120	0.8624	1.1332
89	30	1.002250	4.0	0.70	0.10006	1.16994	2.73009	0.235639	0.236069	0.235976	0.200	1.1443	0.00445	0.8572	1.1342
90	30	1.002250	4.0	0.70	0.10008	1.16994	2.73009	0.235914	0.236344	0.236251	0.200	1.1382	0.00443	0.8572	1.1342
85	40	1.001214	4.0	0.30	0.10022	2.72986	1.17003	0.240289	0.241134	0.241100	0.170	0.9223	0.00307	0.8675	1.1258
86	40	1.001214	4.0	0.30	0.10021	2.72974	1.16997	0.240087	0.240932	0.240898	0.170	0.9259	0.00308	0.8675	1.1258
87	40	1.001214	4.0	0.50	0.10016	1.94991	1.95005	0.235729	0.236568	0.236534	0.200	1.0041	0.00388	0.8622	1.1279
90	40	1.001214	4.0	0.70	0.10008	1.16994	2.73009	0.230786	0.231620	0.231586	0.200	1.1009	0.00425	0.8568	1.1290
91	5	1.008932	4.5	0.30	0.10026	3.07973	1.32007	0.249220	0.249185	0.249189	0.110	1.0863	0.00257	0.8534	1.1570
92	5	1.008932	4.5	0.30	0.10020	3.07987	1.32003	0.249067	0.249032	0.249036	0.110	1.0901	0.00258	0.8534	1.1570
93	5	1.008932	4.5	0.50	0.10014	2.19992	2.20003	0.244575	0.244539	0.244543	0.073	1.1976	0.00195	0.8469	1.1597
94	5	1.008932	4.5	0.50	0.10018	2.19982	2.20002	0.244677	0.244641	0.244645	0.073	1.1948	0.00194	0.8469	1.1597
95	5	1.008932	4.5	0.70	0.10010	1.31994	3.08015	0.239384	0.239348	0.239351	0.010	1.3348	0.00080	0.8404	1.1611
96	5	1.008932	4.5	0.70	0.10012	1.31984	3.08009	0.239383	0.239347	0.239350	0.010	1.3347	0.00080	0.8404	1.1611
91	10	1.006504	4.5	0.30	0.10026	3.07973	1.32007	0.247422	0.247452	0.247431	0.096	1.0719	0.00220	0.8527	1.1543
92	10	1.006504	4.5	0.30	0.10020	3.07987	1.32003	0.247287	0.247317	0.247296	0.096	1.0752	0.00221	0.8527	1.1543
93	10	1.006504	4.5	0.50	0.10014	2.19992	2.20003	0.242694	0.242723	0.242702	0.011	1.1817	0.00073	0.8461	1.1570
94	10	1.006504	4.5	0.50	0.10018	2.19982	2.20002	0.242701	0.242730	0.242709	0.011	1.1813	0.00073	0.8461	1.1570
95	10	1.006504	4.5	0.70	0.10010	1.31994	3.08015	0.237365	0.237393	0.237372	0.010	1.3183	0.00080	0.8395	1.1585
96	10	1.006504	4.5	0.70	0.10012	1.31984	3.08009	0.237365	0.237393	0.237372	0.010	1.3182	0.00080	0.8395	1.1585
91	15	1.006050	4.5	0.30	0.10026	3.07973	1.32007	0.245549	0.245636	0.245622	0.110	1.0559	0.00242	0.8520	1.1516
92	15	1.006050	4.5	0.30	0.10020	3.07987	1.32003	0.245401	0.245488	0.245474	0.110	1.0593	0.00242	0.8520	1.1516
93	15	1.006050	4.5	0.50	0.10014	2.19992	2.20003	0.240707	0.240792	0.240779	0.011	1.1647	0.00071	0.8453	1.1544
94	15	1.006050	4.5	0.50	0.10018	2.19982	2.20002	0.240714	0.240799	0.240786	0.011	1.1644	0.00071	0.8453	1.1544
95	15	1.006050	4.5	0.70	0.10010	1.31994	3.08015	0.235295	0.235379	0.235366	0.011	1.2991	0.00079	0.8387	1.1559
96	15	1.006050	4.5	0.70	0.10012	1.31984	3.08009	0.235292	0.235376	0.235363	0.011	1.2991	0.00079	0.8387	1.1559

91	20	0.999872	4.5	0.30	0.10026	3.07973	1.32007	0.243504	0.243739	0.243699	0.011	1.0399	0.00072	0.8513	1.1489
92	20	0.999872	4.5	0.30	0.10020	3.07987	1.32003	0.243500	0.243735	0.243695	0.011	1.0403	0.00072	0.8513	1.1489
93	20	0.999872	4.5	0.50	0.10014	2.19992	2.20003	0.238584	0.238817	0.238777	0.011	1.1470	0.00079	0.8447	1.1517
94	20	0.999872	4.5	0.50	0.10018	2.19982	2.20002	0.238589	0.238822	0.238782	0.011	1.1466	0.00079	0.8447	1.1517
95	20	0.999872	4.5	0.70	0.10010	1.31994	3.08015	0.233098	0.233329	0.233289	0.011	1.2788	0.00088	0.8381	1.1533
96	20	0.999872	4.5	0.70	0.10012	1.31984	3.08009	0.233094	0.233325	0.233285	0.011	1.2788	0.00088	0.8381	1.1533
91	25	0.997819	4.5	0.30	0.10026	3.07973	1.32007	0.241405	0.241763	0.241699	0.020	1.0226	0.00079	0.8509	1.1461
92	25	0.997819	4.5	0.30	0.10020	3.07987	1.32003	0.241402	0.241760	0.241696	0.020	1.0230	0.00079	0.8509	1.1461
93	25	0.997819	4.5	0.50	0.10014	2.19992	2.20003	0.236385	0.236740	0.236677	0.020	1.1283	0.00087	0.8442	1.1489
94	25	0.997819	4.5	0.50	0.10018	2.19982	2.20002	0.236391	0.236746	0.236683	0.020	1.1279	0.00087	0.8442	1.1489
95	25	0.997819	4.5	0.70	0.10010	1.31994	3.08015	0.230749	0.231101	0.231038	0.020	1.2594	0.00097	0.8376	1.1507
96	25	0.997819	4.5	0.70	0.10012	1.31984	3.08009	0.230753	0.231105	0.231042	0.020	1.2592	0.00097	0.8376	1.1507
91	30	0.995362	4.5	0.30	0.10026	3.07973	1.32007	0.239144	0.239663	0.239571	0.050	1.0062	0.00117	0.8506	1.1432
92	30	0.995362	4.5	0.30	0.10020	3.07987	1.32003	0.239141	0.239660	0.239568	0.050	1.0065	0.00117	0.8506	1.1432
93	30	0.995362	4.5	0.50	0.10014	2.19992	2.20003	0.234036	0.234551	0.234459	0.050	1.1102	0.00129	0.8439	1.1462
94	30	0.995362	4.5	0.50	0.10018	2.19982	2.20002	0.234042	0.234557	0.234465	0.050	1.1099	0.00129	0.8439	1.1462
96	30	0.995362	4.5	0.70	0.10012	1.31984	3.08009	0.228385	0.228897	0.228804	0.180	1.2373	0.00435	0.8373	1.1480
91	40	0.996585	4.5	0.30	0.10026	3.07973	1.32007	0.234275	0.235169	0.235134	0.200	0.9710	0.00375	0.8505	1.1375
93	40	0.996585	4.5	0.50	0.10014	2.19992	2.20003	0.229015	0.229901	0.229867	0.090	1.0712	0.00212	0.8437	1.1406
94	40	0.996585	4.5	0.50	0.10018	2.19982	2.20002	0.229024	0.229910	0.229876	0.090	1.0708	0.00212	0.8437	1.1406
96	40	0.996585	4.5	0.70	0.10012	1.31984	3.08009	0.223208	0.224087	0.224053	0.200	1.1932	0.00461	0.8370	1.1426
97	5	0.999082	5.0	0.30	0.10035	3.42967	1.47001	0.243193	0.243274	0.243278	0.076	1.1653	0.00196	0.8367	1.1690
98	5	0.999082	5.0	0.30	0.10032	3.42990	1.47001	0.243086	0.243167	0.243171	0.076	1.1680	0.00197	0.8367	1.1690
99	5	0.999082	5.0	0.50	0.10021	2.44982	2.45002	0.237837	0.237917	0.237921	0.010	1.3039	0.00078	0.8284	1.1727
100	5	0.999082	5.0	0.50	0.10024	2.44979	2.45005	0.237837	0.237917	0.237921	0.010	1.3037	0.00078	0.8284	1.1727
101	5	0.999082	5.0	0.70	0.10014	1.46982	3.42988	0.231701	0.231781	0.231784	0.010	1.4826	0.00089	0.8202	1.1749
102	5	0.999082	5.0	0.70	0.10013	1.46993	3.43008	0.231703	0.231783	0.231786	0.010	1.4825	0.00089	0.8202	1.1749
97	10	0.998214	5.0	0.30	0.10035	3.42967	1.47001	0.241350	0.241480	0.241458	0.093	1.1488	0.00229	0.8359	1.1662
98	10	0.998214	5.0	0.30	0.10032	3.42990	1.47001	0.241219	0.241349	0.241327	0.093	1.1520	0.00230	0.8359	1.1662
99	10	0.998214	5.0	0.50	0.10021	2.44982	2.45002	0.235890	0.236018	0.235996	0.010	1.2857	0.00078	0.8276	1.1698
100	10	0.998214	5.0	0.50	0.10024	2.44979	2.45005	0.235892	0.236020	0.235998	0.010	1.2855	0.00078	0.8276	1.1698
101	10	0.998214	5.0	0.70	0.10014	1.46982	3.42988	0.229662	0.229789	0.229767	0.010	1.4613	0.00089	0.8193	1.1721
102	10	0.998214	5.0	0.70	0.10013	1.46993	3.43008	0.229664	0.229791	0.229769	0.010	1.4613	0.00089	0.8193	1.1721
97	15	1.000849	5.0	0.30	0.10035	3.42967	1.47001	0.239479	0.239627	0.239614	0.110	1.1300	0.00259	0.8351	1.1632
98	15	1.000849	5.0	0.30	0.10032	3.42990	1.47001	0.239329	0.239477	0.239464	0.110	1.1336	0.00259	0.8351	1.1632

99	15	1.000849	5.0	0.50	0.10021	2.44982	2.45002	0.233912	0.234058	0.234045	0.010	1.2650	0.00076	0.8267	1.1670
100	15	1.000849	5.0	0.50	0.10024	2.44979	2.45005	0.233914	0.234060	0.234047	0.010	1.2648	0.00076	0.8267	1.1670
101	15	1.000849	5.0	0.70	0.10014	1.46982	3.42988	0.227587	0.227732	0.227718	0.010	1.4374	0.00086	0.8185	1.1694
102	15	1.000849	5.0	0.70	0.10013	1.46993	3.43008	0.227588	0.227733	0.227719	0.010	1.4374	0.00086	0.8185	1.1694
97	20	1.003454	5.0	0.30	0.10035	3.42967	1.47001	0.237522	0.237706	0.237666	0.012	1.1112	0.00077	0.8343	1.1603
98	20	1.003454	5.0	0.30	0.10032	3.42990	1.47001	0.237515	0.237699	0.237659	0.012	1.1115	0.00077	0.8343	1.1603
99	20	1.003454	5.0	0.50	0.10021	2.44982	2.45002	0.231882	0.232063	0.232024	0.010	1.2433	0.00085	0.8260	1.1641
100	20	1.003454	5.0	0.50	0.10024	2.44979	2.45005	0.231883	0.232064	0.232025	0.010	1.2431	0.00085	0.8260	1.1641
101	20	1.003454	5.0	0.70	0.10014	1.46982	3.42988	0.225450	0.225629	0.225589	0.010	1.4127	0.00096	0.8178	1.1667
102	20	1.003454	5.0	0.70	0.10013	1.46993	3.43008	0.225452	0.225631	0.225591	0.010	1.4127	0.00096	0.8178	1.1667
97	25	1.001668	5.0	0.30	0.10035	3.42967	1.47001	0.235386	0.235686	0.235622	0.021	1.0914	0.00085	0.8339	1.1573
98	25	1.001668	5.0	0.30	0.10032	3.42990	1.47001	0.235380	0.235680	0.235616	0.021	1.0917	0.00085	0.8339	1.1574
99	25	1.001668	5.0	0.50	0.10021	2.44982	2.45002	0.229648	0.229944	0.229881	0.020	1.2213	0.00094	0.8256	1.1612
100	25	1.001668	5.0	0.50	0.10024	2.44979	2.45005	0.229650	0.229946	0.229883	0.020	1.2211	0.00094	0.8256	1.1612
101	25	1.001668	5.0	0.70	0.10014	1.46982	3.42988	0.222981	0.223274	0.223211	0.140	1.3911	0.00391	0.8173	1.1639
102	25	1.001668	5.0	0.70	0.10013	1.46993	3.43008	0.223175	0.223468	0.223405	0.140	1.3859	0.00389	0.8173	1.1639
97	30	0.999211	5.0	0.30	0.10035	3.42967	1.47001	0.233066	0.233523	0.233431	0.050	1.0731	0.00124	0.8335	1.1544
98	30	0.999211	5.0	0.30	0.10032	3.42990	1.47001	0.233062	0.233519	0.233427	0.050	1.0733	0.00124	0.8335	1.1544
99	30	0.999211	5.0	0.50	0.10021	2.44982	2.45002	0.227260	0.227712	0.227620	0.050	1.2001	0.00139	0.8252	1.1583
100	30	0.999211	5.0	0.50	0.10024	2.44979	2.45005	0.227265	0.227717	0.227625	0.050	1.1999	0.00139	0.8252	1.1583
102	30	0.999211	5.0	0.70	0.10013	1.46993	3.43008	0.220741	0.221189	0.221096	0.180	1.3603	0.00478	0.8170	1.1612
97	40	0.998796	5.0	0.30	0.10035	3.42967	1.47001	0.228077	0.228921	0.228887	0.200	1.0338	0.00399	0.8335	1.1484
99	40	0.998796	5.0	0.50	0.10021	2.44982	2.45002	0.222130	0.222965	0.222931	0.090	1.1552	0.00228	0.8251	1.1526
100	40	0.998796	5.0	0.50	0.10024	2.44979	2.45005	0.222137	0.222972	0.222938	0.090	1.1549	0.00228	0.8251	1.1526
102	40	0.998796	5.0	0.70	0.10013	1.46993	3.43008	0.215535	0.216361	0.216327	0.200	1.3061	0.00504	0.8168	1.1556
103	5	0.993516	5.5	0.30	0.10034	3.77981	1.61999	0.238002	0.238149	0.238152	0.150	1.2365	0.00394	0.8200	1.1803
104	5	0.993516	5.5	0.30	0.10010	3.77057	1.61614	0.238213	0.238360	0.238363	0.150	1.2340	0.00393	0.8205	1.1800
105	5	0.993516	5.5	0.50	0.10026	2.69965	2.70002	0.231874	0.232020	0.232023	0.160	1.4057	0.00477	0.8097	1.1849
106	5	0.993516	5.5	0.50	0.10027	2.69969	2.70005	0.232095	0.232241	0.232244	0.160	1.3991	0.00474	0.8097	1.1849
107	5	0.993516	5.5	0.70	0.10016	1.61984	3.78003	0.224939	0.225084	0.225087	0.150	1.6253	0.00518	0.7996	1.1880
108	5	0.993516	5.5	0.70	0.10015	1.61985	3.77996	0.224731	0.224876	0.224879	0.150	1.6325	0.00520	0.7996	1.1880
103	10	0.991325	5.5	0.30	0.10034	3.77981	1.61999	0.236119	0.236331	0.236309	0.150	1.2173	0.00381	0.8192	1.1772
104	10	0.991325	5.5	0.30	0.10010	3.77057	1.61614	0.236326	0.236538	0.236516	0.150	1.2150	0.00381	0.8196	1.1769
105	10	0.991325	5.5	0.50	0.10026	2.69965	2.70002	0.229886	0.230097	0.230075	0.150	1.3837	0.00433	0.8089	1.1819
106	10	0.991325	5.5	0.50	0.10027	2.69969	2.70005	0.230103	0.230314	0.230292	0.150	1.3775	0.00431	0.8089	1.1819

107	10	0.991325	5.5	0.70	0.10016	1.61984	3.78003	0.222844	0.223053	0.223031	0.130	1.5994	0.00436	0.7987	1.1851
108	10	0.991325	5.5	0.70	0.10015	1.61985	3.77996	0.222659	0.222868	0.222846	0.130	1.6055	0.00438	0.7987	1.1851
103	15	0.993032	5.5	0.30	0.10034	3.77981	1.61999	0.234205	0.234448	0.234435	0.130	1.1959	0.00321	0.8182	1.1741
104	15	0.993032	5.5	0.30	0.10010	3.77057	1.61614	0.234386	0.234629	0.234616	0.130	1.1944	0.00320	0.8187	1.1738
105	15	0.993032	5.5	0.50	0.10026	2.69965	2.70002	0.227878	0.228119	0.228106	0.140	1.3590	0.00391	0.8079	1.1788
106	15	0.993032	5.5	0.50	0.10027	2.69969	2.70005	0.228079	0.228320	0.228307	0.140	1.3535	0.00390	0.8079	1.1788
107	15	0.993032	5.5	0.70	0.10016	1.61984	3.78003	0.220729	0.220968	0.220954	0.085	1.5703	0.00283	0.7978	1.1823
108	15	0.993032	5.5	0.70	0.10015	1.61985	3.77996	0.220610	0.220849	0.220835	0.085	1.5742	0.00284	0.7978	1.1823
103	20	0.996398	5.5	0.30	0.10034	3.77981	1.61999	0.232244	0.232514	0.232474	0.074	1.1742	0.00188	0.8173	1.1710
104	20	0.996398	5.5	0.30	0.10010	3.77057	1.61614	0.232348	0.232618	0.232578	0.074	1.1746	0.00189	0.8177	1.1707
105	20	0.996398	5.5	0.50	0.10026	2.69965	2.70002	0.225833	0.226099	0.226060	0.120	1.3337	0.00329	0.8071	1.1758
106	20	0.996398	5.5	0.50	0.10027	2.69969	2.70005	0.225999	0.226265	0.226226	0.120	1.3292	0.00328	0.8071	1.1758
107	20	0.996398	5.5	0.70	0.10016	1.61984	3.78003	0.218583	0.218846	0.218807	0.011	1.5403	0.00106	0.7971	1.1794
108	20	0.996398	5.5	0.70	0.10015	1.61985	3.77996	0.218579	0.218842	0.218803	0.011	1.5405	0.00106	0.7971	1.1794
103	25	0.994503	5.5	0.30	0.10034	3.77981	1.61999	0.230077	0.230464	0.230401	0.079	1.1520	0.00193	0.8168	1.1679
104	25	0.994503	5.5	0.30	0.10010	3.77057	1.61614	0.230184	0.230571	0.230508	0.079	1.1524	0.00193	0.8173	1.1676
105	25	0.994503	5.5	0.50	0.10026	2.69965	2.70002	0.223573	0.223956	0.223893	0.120	1.3081	0.00318	0.8067	1.1728
106	25	0.994503	5.5	0.50	0.10027	2.69969	2.70005	0.223737	0.224120	0.224057	0.120	1.3039	0.00317	0.8067	1.1728
107	25	0.994503	5.5	0.70	0.10016	1.61984	3.78003	0.216101	0.216480	0.216416	0.088	1.5137	0.00278	0.7967	1.1766
108	25	0.994503	5.5	0.70	0.10015	1.61985	3.77996	0.216223	0.216602	0.216538	0.088	1.5102	0.00278	0.7967	1.1766
103	30	0.993901	5.5	0.30	0.10034	3.77981	1.61999	0.227812	0.228331	0.228239	0.051	1.1301	0.00133	0.8165	1.1648
104	30	0.993901	5.5	0.30	0.10010	3.77057	1.61614	0.227821	0.228340	0.228248	0.051	1.1326	0.00133	0.8169	1.1645
105	30	0.993901	5.5	0.50	0.10026	2.69965	2.70002	0.221167	0.221680	0.221588	0.150	1.2840	0.00379	0.8064	1.1698
106	30	0.993901	5.5	0.50	0.10027	2.69969	2.70005	0.221370	0.221883	0.221791	0.150	1.2790	0.00377	0.8064	1.1698
108	30	0.993901	5.5	0.70	0.10015	1.61985	3.77996	0.213874	0.214382	0.214289	0.180	1.4773	0.00519	0.7964	1.1737
103	40	0.996082	5.5	0.30	0.10034	3.77981	1.61999	0.222936	0.223801	0.223767	0.090	1.0838	0.00214	0.8165	1.1586
104	40	0.996082	5.5	0.30	0.10010	3.77057	1.61614	0.222935	0.223800	0.223766	0.090	1.0864	0.00215	0.8170	1.1584
105	40	0.996082	5.5	0.50	0.10026	2.69965	2.70002	0.216084	0.216938	0.216904	0.190	1.2313	0.00453	0.8064	1.1639
106	40	0.996082	5.5	0.50	0.10027	2.69969	2.70005	0.216326	0.217180	0.217146	0.190	1.2257	0.00451	0.8064	1.1639

[a] Columns $m\mathrm{Cl}^-$ and $y\mathrm{Na}^+$ contain rounded values, and exact values can be calculated from the listed *m*HCl, *m*TrisHCl, and *m*NaCl. Cell potentials and their uncertainties are printed to fixed numbers of digits following the decimal point, for reasons of simplic ity.

[b] Measurement temperatures are equal to 5.000 °C, 10.000 °C, etc. within the expanded uncertainty of the thermometer (±0.007 °C, $k = 2$ from the calibration certificate), and are presented here as integer values.

[c] The meanings of the listed potentials are as follows: *E*(meas.) – the unadjusted measured values at the listed pressure, *P*; *E* – measured potentials corrected to 1 atm pH_2; *E*(adj.) – values of *E* adjusted to the standard potentials of Bates and Bower,[13] as noted in section 3.1 of the main text.

Table S7. Harned cell results for HCl-NaCl-TrisHCl solutions, for ionic strengths of 2.0 mol kg^{-1} and below [a]

Cell	*t* (°C)	*P* (atm)	*m*Cl$^-$ (mol kg^{-1})	*y*Na$^+$	*m*HCl (mol kg^{-1})	*m*TrisHCl (mol kg^{-1})	*m*NaCl (mol kg^{-1})	*E*(meas.) (V)	*E* (V)	*E*(adj.) (V)	u(*E*) (mV)	γ_{HCl}	u(γ_{HCl})	*a*H_2O	ρ (g cm^{-3})
1	5	1.003750	1.0	0.30	0.10000	0.62997	0.26993	0.303393	0.303434	0.303339	0.012	0.7457	0.00046	0.9677	1.0432
2	5	1.003750	1.0	0.30	0.10001	0.63004	0.26995	0.303401	0.303441	0.303347	0.012	0.7455	0.00046	0.9677	1.0432
3	5	1.003750	1.0	0.50	0.10002	0.44999	0.45001	0.302924	0.302964	0.302869	0.013	0.7529	0.00047	0.9674	1.0424
4	5	1.003750	1.0	0.50	0.10024	0.44997	0.44998	0.302914	0.302954	0.302859	0.013	0.7522	0.00047	0.9674	1.0424
5	5	1.003750	1.0	0.70	0.10010	0.27001	0.62991	0.302155	0.302195	0.302100	0.011	0.7648	0.00047	0.9672	1.0413
6	5	1.003750	1.0	0.70	0.09993	0.27002	0.62997	0.302159	0.302199	0.302104	0.011	0.7654	0.00047	0.9672	1.0413
1	10	1.002675	1.0	0.30	0.10000	0.62997	0.26993	0.302172	0.302268	0.302132	0.072	0.7422	0.00118	0.9676	1.0424
2	10	1.002675	1.0	0.30	0.10001	0.63004	0.26995	0.302273	0.302369	0.302233	0.072	0.7406	0.00117	0.9676	1.0424
3	10	1.002675	1.0	0.50	0.10002	0.44999	0.45001	0.301667	0.301763	0.301627	0.073	0.7498	0.00120	0.9673	1.0415
4	10	1.002675	1.0	0.50	0.10024	0.44997	0.44998	0.301565	0.301661	0.301525	0.073	0.7505	0.00120	0.9673	1.0415
5	10	1.002675	1.0	0.70	0.10010	0.27001	0.62991	0.300846	0.300942	0.300806	0.010	0.7623	0.00046	0.9671	1.0404
6	10	1.002675	1.0	0.70	0.09993	0.27002	0.62997	0.300848	0.300944	0.300808	0.010	0.7629	0.00046	0.9671	1.0404
1	15	1.005270	1.0	0.30	0.10000	0.62997	0.26993	0.300853	0.300975	0.300836	0.077	0.7378	0.00122	0.9676	1.0413
2	15	1.005270	1.0	0.30	0.10001	0.63004	0.26995	0.300961	0.301083	0.300944	0.077	0.7362	0.00122	0.9676	1.0413
3	15	1.005270	1.0	0.50	0.10002	0.44999	0.45001	0.300315	0.300437	0.300298	0.013	0.7458	0.00046	0.9673	1.0403
4	15	1.005270	1.0	0.50	0.10024	0.44997	0.44998	0.300305	0.300427	0.300288	0.013	0.7450	0.00046	0.9673	1.0403
5	15	1.005270	1.0	0.70	0.10010	0.27001	0.62991	0.299466	0.299588	0.299449	0.010	0.7583	0.00045	0.9670	1.0392
6	15	1.005270	1.0	0.70	0.09993	0.27002	0.62997	0.299467	0.299589	0.299450	0.010	0.7590	0.00045	0.9670	1.0392
1	20	1.013047	1.0	0.30	0.10000	0.62997	0.26993	0.299465	0.299567	0.299375	0.085	0.7338	0.00133	0.9676	1.0399
2	20	1.013047	1.0	0.30	0.10001	0.63004	0.26995	0.299578	0.299681	0.299488	0.085	0.7321	0.00132	0.9676	1.0399
3	20	1.013047	1.0	0.50	0.10002	0.44999	0.45001	0.298881	0.298983	0.298791	0.012	0.7423	0.00052	0.9672	1.0390
4	20	1.013047	1.0	0.50	0.10024	0.44997	0.44998	0.298872	0.298974	0.298782	0.012	0.7415	0.00052	0.9672	1.0390
5	20	1.013047	1.0	0.70	0.10010	0.27001	0.62991	0.298019	0.298121	0.297929	0.010	0.7547	0.00052	0.9669	1.0378
6	20	1.013047	1.0	0.70	0.09993	0.27002	0.62997	0.298020	0.298122	0.297930	0.010	0.7554	0.00052	0.9669	1.0378
1	25	1.012218	1.0	0.30	0.10000	0.62997	0.26993	0.297823	0.298041	0.297830	0.082	0.7286	0.00126	0.9675	1.0384
2	25	1.012218	1.0	0.30	0.10001	0.63004	0.26995	0.297935	0.298153	0.297942	0.082	0.7270	0.00126	0.9675	1.0384

3	25	1.012218	1.0	0.50	0.10002	0.44999	0.45001	0.297204	0.297422	0.297211	0.021	0.7373	0.00057	0.9672	1.0374
4	25	1.012218	1.0	0.50	0.10024	0.44997	0.44998	0.297195	0.297413	0.297202	0.021	0.7366	0.00057	0.9672	1.0374
5	25	1.012218	1.0	0.70	0.10010	0.27001	0.62991	0.296304	0.296522	0.296311	0.020	0.7501	0.00058	0.9669	1.0363
6	25	1.012218	1.0	0.70	0.09993	0.27002	0.62997	0.296304	0.296522	0.296311	0.020	0.7507	0.00058	0.9669	1.0363
1	30	1.009731	1.0	0.30	0.10000	0.62997	0.26993	0.296014	0.296407	0.296109	0.097	0.7243	0.00143	0.9675	1.0367
2	30	1.009731	1.0	0.30	0.10001	0.63004	0.26995	0.296131	0.296524	0.296226	0.097	0.7226	0.00142	0.9675	1.0367
3	30	1.009731	1.0	0.50	0.10002	0.44999	0.45001	0.295368	0.295760	0.295463	0.050	0.7332	0.00085	0.9672	1.0357
4	30	1.009731	1.0	0.50	0.10024	0.44997	0.44998	0.295359	0.295752	0.295454	0.050	0.7324	0.00085	0.9672	1.0357
5	30	1.009731	1.0	0.70	0.10010	0.27001	0.62991	0.294445	0.294837	0.294540	0.050	0.7459	0.00086	0.9668	1.0345
6	30	1.009731	1.0	0.70	0.09993	0.27002	0.62997	0.294445	0.294837	0.294540	0.050	0.7466	0.00086	0.9668	1.0345
1	40	1.009099	1.0	0.30	0.10000	0.62997	0.26993	0.291804	0.292642	0.292648	0.120	0.7106	0.00171	0.9676	1.0328
2	40	1.009099	1.0	0.30	0.10001	0.63004	0.26995	0.291918	0.292756	0.292762	0.120	0.7090	0.00170	0.9676	1.0328
3	40	1.009099	1.0	0.50	0.10002	0.44999	0.45001	0.291111	0.291949	0.291955	0.120	0.7197	0.00173	0.9672	1.0318
4	40	1.009099	1.0	0.50	0.10024	0.44997	0.44998	0.291003	0.291841	0.291847	0.120	0.7202	0.00173	0.9672	1.0318
5	40	1.009099	1.0	0.70	0.10010	0.27001	0.62991	0.290126	0.290963	0.290969	0.090	0.7326	0.00139	0.9668	1.0306
6	40	1.009099	1.0	0.70	0.09993	0.27002	0.62997	0.290124	0.290961	0.290967	0.090	0.7333	0.00139	0.9668	1.0306
7	5	1.011093	1.5	0.30	0.09990	0.98005	0.41996	0.291959	0.291910	0.291815	0.110	0.7747	0.00183	0.9517	1.0635
8	5	1.011093	1.5	0.30	0.09994	0.98001	0.42001	0.292112	0.292063	0.291968	0.110	0.7721	0.00183	0.9517	1.0635
9	5	1.011093	1.5	0.50	0.09995	0.69999	0.70001	0.290961	0.290911	0.290817	0.011	0.7908	0.00048	0.9512	1.0626
10	5	1.011093	1.5	0.50	0.09998	0.69998	0.70002	0.290956	0.290907	0.290812	0.011	0.7908	0.00048	0.9512	1.0626
11	5	1.011093	1.5	0.70	0.10000	0.42001	0.97997	0.289791	0.289741	0.289647	0.010	0.8101	0.00049	0.9506	1.0613
12	5	1.011093	1.5	0.70	0.10002	0.42000	0.97998	0.289794	0.289745	0.289650	0.010	0.8100	0.00049	0.9506	1.0613
7	10	1.007205	1.5	0.30	0.09990	0.98005	0.41996	0.290560	0.290598	0.290462	0.110	0.7701	0.00179	0.9516	1.0624
8	10	1.007205	1.5	0.30	0.09994	0.98001	0.42001	0.290711	0.290749	0.290613	0.110	0.7676	0.00179	0.9516	1.0624
9	10	1.007205	1.5	0.50	0.09995	0.69999	0.70001	0.289510	0.289548	0.289412	0.011	0.7867	0.00049	0.9510	1.0614
10	10	1.007205	1.5	0.50	0.09998	0.69998	0.70002	0.289505	0.289543	0.289407	0.011	0.7866	0.00049	0.9510	1.0614
11	10	1.007205	1.5	0.70	0.10000	0.42001	0.97997	0.288267	0.288305	0.288169	0.011	0.8068	0.00050	0.9503	1.0601
12	10	1.007205	1.5	0.70	0.10002	0.42000	0.97998	0.288272	0.288310	0.288174	0.011	0.8066	0.00050	0.9503	1.0601
7	15	1.004648	1.5	0.30	0.09990	0.98005	0.41996	0.289055	0.289181	0.289042	0.110	0.7643	0.00175	0.9515	1.0610
8	15	1.004648	1.5	0.30	0.09994	0.98001	0.42001	0.289209	0.289336	0.289196	0.110	0.7618	0.00174	0.9515	1.0610
9	15	1.004648	1.5	0.50	0.09995	0.69999	0.70001	0.287963	0.288089	0.287950	0.011	0.7811	0.00047	0.9508	1.0600
10	15	1.004648	1.5	0.50	0.09998	0.69998	0.70002	0.287957	0.288083	0.287944	0.011	0.7811	0.00047	0.9508	1.0600
11	15	1.004648	1.5	0.70	0.10000	0.42001	0.97997	0.286653	0.286779	0.286640	0.011	0.8018	0.00049	0.9502	1.0587
12	15	1.004648	1.5	0.70	0.10002	0.42000	0.97998	0.286657	0.286783	0.286644	0.011	0.8016	0.00049	0.9502	1.0587
7	20	1.003365	1.5	0.30	0.09990	0.98005	0.41996	0.287420	0.287641	0.287449	0.120	0.7591	0.00187	0.9514	1.0594

8	20	1.003365	1.5	0.30	0.09994	0.98001	0.42001	0.287590	0.287811	0.287619	0.120	0.7564	0.00187	0.9514	1.0594
9	20	1.003365	1.5	0.50	0.09995	0.69999	0.70001	0.286284	0.286505	0.286313	0.011	0.7761	0.00053	0.9507	1.0584
10	20	1.003365	1.5	0.50	0.09998	0.69998	0.70002	0.286278	0.286499	0.286307	0.011	0.7761	0.00053	0.9507	1.0584
11	20	1.003365	1.5	0.70	0.10000	0.42001	0.97997	0.284946	0.285167	0.284975	0.011	0.7967	0.00055	0.9500	1.0571
12	20	1.003365	1.5	0.70	0.10002	0.42000	0.97998	0.284949	0.285170	0.284978	0.011	0.7966	0.00055	0.9500	1.0571
7	25	1.002536	1.5	0.30	0.09990	0.98005	0.41996	0.285653	0.285991	0.285780	0.100	0.7525	0.00155	0.9514	1.0577
8	25	1.002536	1.5	0.30	0.09994	0.98001	0.42001	0.285796	0.286134	0.285923	0.100	0.7503	0.00154	0.9514	1.0577
9	25	1.002536	1.5	0.50	0.09995	0.69999	0.70001	0.284480	0.284818	0.284607	0.021	0.7697	0.00060	0.9506	1.0567
10	25	1.002536	1.5	0.50	0.09998	0.69998	0.70002	0.284473	0.284811	0.284600	0.021	0.7697	0.00060	0.9506	1.0567
11	25	1.002536	1.5	0.70	0.10000	0.42001	0.97997	0.283077	0.283415	0.283203	0.020	0.7908	0.00061	0.9499	1.0554
12	25	1.002536	1.5	0.70	0.10002	0.42000	0.97998	0.283080	0.283418	0.283206	0.020	0.7907	0.00061	0.9499	1.0554
7	30	0.999724	1.5	0.30	0.09990	0.98005	0.41996	0.283719	0.284238	0.283940	0.120	0.7468	0.00178	0.9513	1.0558
8	30	0.999724	1.5	0.30	0.09994	0.98001	0.42001	0.283873	0.284392	0.284094	0.120	0.7444	0.00178	0.9513	1.0558
9	30	0.999724	1.5	0.50	0.09995	0.69999	0.70001	0.282501	0.283019	0.282722	0.050	0.7642	0.00088	0.9506	1.0548
10	30	0.999724	1.5	0.50	0.09998	0.69998	0.70002	0.282495	0.283013	0.282716	0.050	0.7642	0.00088	0.9506	1.0548
11	30	0.999724	1.5	0.70	0.10000	0.42001	0.97997	0.281057	0.281575	0.281277	0.050	0.7854	0.00091	0.9498	1.0535
12	30	0.999724	1.5	0.70	0.10002	0.42000	0.97998	0.281061	0.281579	0.281281	0.050	0.7853	0.00091	0.9498	1.0535
7	40	0.998026	1.5	0.30	0.09990	0.98005	0.41996	0.279276	0.280256	0.280262	0.140	0.7302	0.00201	0.9513	1.0516
8	40	0.998026	1.5	0.30	0.09994	0.98001	0.42001	0.279420	0.280400	0.280406	0.140	0.7281	0.00200	0.9513	1.0516
9	40	0.998026	1.5	0.50	0.09995	0.69999	0.70001	0.277982	0.278962	0.278968	0.090	0.7477	0.00142	0.9505	1.0506
10	40	0.998026	1.5	0.50	0.09998	0.69998	0.70002	0.277975	0.278955	0.278961	0.090	0.7477	0.00142	0.9505	1.0506
11	40	0.998026	1.5	0.70	0.10000	0.42001	0.97997	0.276455	0.277434	0.277440	0.090	0.7690	0.00146	0.9497	1.0493
12	40	0.998026	1.5	0.70	0.10002	0.42000	0.97998	0.276461	0.277440	0.277446	0.090	0.7688	0.00146	0.9497	1.0493
13	5	1.004619	2.0	0.30	0.10006	1.33000	0.56996	0.282285	0.282311	0.282216	0.140	0.8190	0.00244	0.9357	1.0822
14	5	1.004619	2.0	0.30	0.10004	1.32995	0.56999	0.282483	0.282509	0.282414	0.140	0.8157	0.00243	0.9357	1.0822
15	5	1.004619	2.0	0.50	0.10009	0.94979	0.95026	0.280765	0.280791	0.280696	0.160	0.8452	0.00287	0.9346	1.0815
16	5	1.004619	2.0	0.50	0.10001	0.95002	0.94994	0.280985	0.281011	0.280916	0.160	0.8417	0.00285	0.9346	1.0815
17	5	1.004619	2.0	0.70	0.10000	0.57000	1.32998	0.279349	0.279375	0.279280	0.140	0.8710	0.00259	0.9335	1.0803
18	5	1.004619	2.0	0.70	0.09995	0.57006	1.32999	0.279158	0.279184	0.279089	0.140	0.8747	0.00261	0.9335	1.0803
13	10	1.002013	2.0	0.30	0.10006	1.33000	0.56996	0.280806	0.280905	0.280769	0.140	0.8128	0.00238	0.9355	1.0808
14	10	1.002013	2.0	0.30	0.10004	1.32995	0.56999	0.280997	0.281096	0.280960	0.140	0.8097	0.00237	0.9355	1.0808
15	10	1.002013	2.0	0.50	0.10009	0.94979	0.95026	0.279194	0.279293	0.279157	0.160	0.8400	0.00280	0.9343	1.0800
16	10	1.002013	2.0	0.50	0.10001	0.95002	0.94994	0.279417	0.279516	0.279380	0.160	0.8365	0.00279	0.9343	1.0800
17	10	1.002013	2.0	0.70	0.10000	0.57000	1.32998	0.277708	0.277807	0.277671	0.140	0.8664	0.00254	0.9332	1.0788
18	10	1.002013	2.0	0.70	0.09995	0.57006	1.32999	0.277515	0.277614	0.277478	0.140	0.8700	0.00255	0.9332	1.0788

13	15	1.001757	2.0	0.30	0.10006	1.33000	0.56996	0.279221	0.279380	0.279240	0.140	0.8057	0.00232	0.9353	1.0792
14	15	1.001757	2.0	0.30	0.10004	1.32995	0.56999	0.279413	0.279572	0.279432	0.140	0.8026	0.00231	0.9353	1.0792
15	15	1.001757	2.0	0.50	0.10009	0.94979	0.95026	0.277535	0.277694	0.277554	0.170	0.8333	0.00290	0.9341	1.0784
16	15	1.001757	2.0	0.50	0.10001	0.95002	0.94994	0.277771	0.277930	0.277790	0.170	0.8297	0.00288	0.9341	1.0784
17	15	1.001757	2.0	0.70	0.10000	0.57000	1.32998	0.275986	0.276145	0.276005	0.140	0.8602	0.00248	0.9329	1.0771
18	15	1.001757	2.0	0.70	0.09995	0.57006	1.32999	0.275782	0.275941	0.275801	0.140	0.8639	0.00249	0.9329	1.0771
13	20	1.005319	2.0	0.30	0.10006	1.33000	0.56996	0.277555	0.277746	0.277554	0.150	0.7989	0.00243	0.9351	1.0774
14	20	1.005319	2.0	0.30	0.10004	1.32995	0.56999	0.277766	0.277957	0.277765	0.150	0.7957	0.00242	0.9351	1.0774
15	20	1.005319	2.0	0.50	0.10009	0.94979	0.95026	0.275810	0.276001	0.275808	0.170	0.8269	0.00284	0.9339	1.0766
16	20	1.005319	2.0	0.50	0.10001	0.95002	0.94994	0.276044	0.276235	0.276042	0.170	0.8234	0.00283	0.9339	1.0766
17	20	1.005319	2.0	0.70	0.10000	0.57000	1.32998	0.274192	0.274382	0.274190	0.150	0.8542	0.00260	0.9326	1.0753
18	20	1.005319	2.0	0.70	0.09995	0.57006	1.32999	0.273974	0.274164	0.273972	0.150	0.8581	0.00261	0.9326	1.0753
13	25	1.004826	2.0	0.30	0.10006	1.33000	0.56996	0.275714	0.276015	0.275804	0.020	0.7907	0.00061	0.9350	1.0754
14	25	1.004826	2.0	0.30	0.10004	1.32995	0.56999	0.275717	0.276018	0.275807	0.020	0.7907	0.00061	0.9350	1.0754
15	25	1.004826	2.0	0.50	0.10009	0.94979	0.95026	0.273902	0.274203	0.273991	0.170	0.8189	0.00277	0.9337	1.0747
16	25	1.004826	2.0	0.50	0.10001	0.95002	0.94994	0.274138	0.274439	0.274227	0.170	0.8155	0.00276	0.9337	1.0747
17	25	1.004826	2.0	0.70	0.10000	0.57000	1.32998	0.272216	0.272516	0.272305	0.150	0.8466	0.00254	0.9324	1.0734
18	25	1.004826	2.0	0.70	0.09995	0.57006	1.32999	0.272001	0.272301	0.272090	0.150	0.8504	0.00255	0.9324	1.0734
13	30	1.002240	2.0	0.30	0.10006	1.33000	0.56996	0.273699	0.274174	0.273877	0.160	0.7835	0.00246	0.9350	1.0734
14	30	1.002240	2.0	0.30	0.10004	1.32995	0.56999	0.273914	0.274389	0.274092	0.160	0.7804	0.00245	0.9350	1.0734
15	30	1.002240	2.0	0.50	0.10009	0.94979	0.95026	0.271843	0.272317	0.272020	0.180	0.8117	0.00285	0.9336	1.0726
16	30	1.002240	2.0	0.50	0.10001	0.95002	0.94994	0.272086	0.272560	0.272263	0.180	0.8083	0.00284	0.9336	1.0726
17	30	1.002240	2.0	0.70	0.10000	0.57000	1.32998	0.270112	0.270585	0.270288	0.170	0.8394	0.00279	0.9323	1.0714
18	30	1.002240	2.0	0.70	0.09995	0.57006	1.32999	0.269879	0.270352	0.270055	0.170	0.8434	0.00280	0.9323	1.0714
13	40	1.000780	2.0	0.30	0.10006	1.33000	0.56996	0.269097	0.270020	0.270026	0.190	0.7638	0.00278	0.9349	1.0690
14	40	1.000780	2.0	0.30	0.10004	1.32995	0.56999	0.269337	0.270260	0.270266	0.190	0.7605	0.00277	0.9349	1.0690
15	40	1.000780	2.0	0.50	0.10009	0.94979	0.95026	0.267158	0.268080	0.268086	0.200	0.7916	0.00303	0.9335	1.0682
16	40	1.000780	2.0	0.50	0.10001	0.95002	0.94994	0.267414	0.268336	0.268342	0.200	0.7882	0.00301	0.9335	1.0682
17	40	1.000780	2.0	0.70	0.10000	0.57000	1.32998	0.265342	0.266262	0.266268	0.200	0.8191	0.00313	0.9321	1.0670
18	40	1.000780	2.0	0.70	0.09995	0.57006	1.32999	0.265098	0.266018	0.266024	0.200	0.8231	0.00315	0.9321	1.0670

[a] The meanings of all the headers, and notes concerning the table content, are the same as for Table S6 above. Cell potentials and their uncertainties are printed to fixed numbers of digits following the decimal point, for reasons of simplicity.

Table S8: Harned Cell Results For 0.04 mol kg^{-1} Tris Buffer in Aqueous NaCl Solutions at Ionic Strengths of 0.2, 1.0, and 4.0 mol kg^{-1}, Including Values of the Acidity Function (eq 5) [a]

Cell	t (°C)	P (atm)	mCl$^-$ (mol kg^{-1})	mTris (mol kg^{-1})	mTrisHCl (mol kg^{-1})	mNaCl (mol kg^{-1})	E(meas.) (V)	E (V)	E(adj.) (V)	u(E) (mV)	Acidity function Q [b]	u(Q)	aH$_2$O	ρ (g cm^{-3})
73	5	0.995776	0.20	0.03998	0.04004	0.16000	0.767767	0.767907	0.767910	0.096	-20.6624	0.0042	0.9927	1.0102
74	5	0.995776	0.20	0.04001	0.04001	0.16000	0.767634	0.767774	0.767777	0.096	-20.6567	0.0042	0.9927	1.0102
75	5	0.995776	1.00	0.04001	0.04000	0.96004	0.737438	0.737575	0.737578	0.020	-21.0062	0.0014	0.9667	1.0428
76	5	0.995776	1.00	0.04001	0.04001	0.96001	0.737434	0.737571	0.737574	0.020	-21.0060	0.0014	0.9667	1.0428
77	5	0.995776	4.00	0.04001	0.03999	3.96013	0.707052	0.707175	0.707179	0.150	-21.1242	0.0064	0.8540	1.1470
78	5	0.995776	4.00	0.03995	0.04003	3.96006	0.706843	0.706966	0.706970	0.150	-21.1155	0.0064	0.8540	1.1470
73	10	0.993930	0.20	0.03998	0.04004	0.16000	0.765626	0.765834	0.765812	0.098	-20.2930	0.0042	0.9927	1.0098
74	10	0.993930	0.20	0.04001	0.04001	0.16000	0.765490	0.765698	0.765676	0.098	-20.2872	0.0042	0.9927	1.0098
75	10	0.993930	1.00	0.04001	0.04000	0.96004	0.734672	0.734876	0.734854	0.021	-20.6334	0.0014	0.9665	1.0418
76	10	0.993930	1.00	0.04001	0.04001	0.96001	0.734665	0.734869	0.734847	0.021	-20.6331	0.0014	0.9665	1.0418
77	10	0.993930	4.00	0.04001	0.03999	3.96013	0.703538	0.703723	0.703701	0.140	-20.7430	0.0059	0.8530	1.1448
78	10	0.993930	4.00	0.03995	0.04003	3.96006	0.703347	0.703532	0.703510	0.140	-20.7351	0.0059	0.8530	1.1448
73	15	0.995588	0.20	0.03998	0.04004	0.16000	0.763443	0.763693	0.763680	0.110	-19.9414	0.0046	0.9927	1.0090
74	15	0.995588	0.20	0.04001	0.04001	0.16000	0.763293	0.763543	0.763530	0.110	-19.9352	0.0046	0.9927	1.0090
75	15	0.995588	1.00	0.04001	0.04000	0.96004	0.731872	0.732116	0.732103	0.021	-20.2790	0.0014	0.9664	1.0406
76	15	0.995588	1.00	0.04001	0.04001	0.96001	0.731862	0.732106	0.732093	0.021	-20.2786	0.0014	0.9664	1.0406
77	15	0.995588	4.00	0.04001	0.03999	3.96013	0.700006	0.700224	0.700211	0.110	-20.3809	0.0046	0.8521	1.1425
78	15	0.995588	4.00	0.03995	0.04003	3.96006	0.699854	0.700072	0.700059	0.110	-20.3748	0.0046	0.8521	1.1425
73	20	1.001332	0.20	0.03998	0.04004	0.16000	0.761230	0.761490	0.761451	0.110	-19.6039	0.0045	0.9927	1.0080
74	20	1.001332	0.20	0.04001	0.04001	0.16000	0.761080	0.761340	0.761301	0.110	-19.5979	0.0045	0.9927	1.0080
75	20	1.001332	1.00	0.04001	0.04000	0.96004	0.729061	0.729313	0.729273	0.130	-19.9394	0.0053	0.9663	1.0392
76	20	1.001332	1.00	0.04001	0.04001	0.96001	0.728873	0.729125	0.729085	0.130	-19.9320	0.0053	0.9663	1.0392
77	20	1.001332	4.00	0.04001	0.03999	3.96013	0.696529	0.696746	0.696706	0.100	-20.0365	0.0042	0.8514	1.1402
78	20	1.001332	4.00	0.03995	0.04003	3.96006	0.696390	0.696607	0.696567	0.100	-20.0310	0.0042	0.8514	1.1402
73	25	1.001579	0.20	0.03998	0.04004	0.16000	0.758819	0.759188	0.759124	0.110	-19.2810	0.0045	0.9927	1.0067
74	25	1.001579	0.20	0.04001	0.04001	0.16000	0.758667	0.759036	0.758972	0.110	-19.2750	0.0045	0.9927	1.0067
75	25	1.001579	1.00	0.04001	0.04000	0.96004	0.726084	0.726441	0.726378	0.140	-19.6157	0.0056	0.9662	1.0376
76	25	1.001579	1.00	0.04001	0.04001	0.96001	0.725887	0.726244	0.726181	0.140	-19.6081	0.0056	0.9662	1.0376
77	25	1.001579	4.00	0.04001	0.03999	3.96013	0.692873	0.693181	0.693118	0.067	-19.7075	0.0029	0.8508	1.1378
78	25	1.001579	4.00	0.03995	0.04003	3.96006	0.692864	0.693172	0.693109	0.067	-19.7071	0.0029	0.8508	1.1378

73	30	1.001135	0.20	0.03998	0.04004	0.16000	0.756284	0.756808	0.756716	0.110	-18.9706	0.0044	0.9927	1.0052
74	30	1.001135	0.20	0.04001	0.04001	0.16000	0.756130	0.756654	0.756562	0.110	-18.9646	0.0044	0.9927	1.0052
75	30	1.001135	1.00	0.04001	0.04000	0.96004	0.722992	0.723500	0.723408	0.150	-19.3048	0.0059	0.9661	1.0358
76	30	1.001135	1.00	0.04001	0.04001	0.96001	0.722776	0.723284	0.723192	0.150	-19.2966	0.0059	0.9661	1.0358
77	30	1.001135	4.00	0.04001	0.03999	3.96013	0.688955	0.689396	0.689304	0.100	-19.3856	0.0040	0.8503	1.1353
78	30	1.001135	4.00	0.03995	0.04003	3.96006	0.689096	0.689537	0.689445	0.100	-19.3910	0.0040	0.8503	1.1353
73	40	1.002369	0.20	0.03998	0.04004	0.16000	0.750876	0.751838	0.751804	0.110	-18.3919	0.0046	0.9927	1.0017
74	40	1.002369	0.20	0.04001	0.04001	0.16000	0.750724	0.751686	0.751652	0.110	-18.3861	0.0046	0.9927	1.0017
75	40	1.002369	1.00	0.04001	0.04000	0.96004	0.716539	0.717472	0.717438	0.170	-18.7277	0.0066	0.9660	1.0318
76	40	1.002369	1.00	0.04001	0.04001	0.96001	0.716299	0.717232	0.717198	0.170	-18.7188	0.0066	0.9660	1.0318
77	40	1.002369	4.00	0.04001	0.03999	3.96013	0.680818	0.681628	0.681593	0.490	-18.7856	0.0183	0.8498	1.1302
78	40	1.002369	4.00	0.03995	0.04003	3.96006	0.681506	0.682316	0.682281	0.490	-18.8111	0.0183	0.8498	1.1302

[a] The meanings of the headers, and notes concerning the table content, are the same as for Table S6 above. Cell potentials and values of Q, and their uncertainties, are printed to fixed numbers of digits following the decimal point, for reasons of simplicity.

[b] The acidity function Q is equal to $\ln(m\mathrm{H}^{+}\cdot\gamma_{\mathrm{HCl}}^{2})$, see eq 5.

References

1. Maksimov, I.; Asakai, T.; Hibino, Y.; Clegg, S. L., Activity coefficients of HCl in solutions related to 'Tris' Buffers in artificial seawater. I. HCl + TrisHCl + H_2O from 1.0 to 5.0 mol kg^{-1} ionic strength, and from 5 °C to 45 °C. *J. Chem. Eng. Data* **2025**, *70*, 1994-2005.

2. Joint Committee for Guides in Metrology (2008) *Evaluation of measurement data — Guide to the expression of uncertainty in measurement*. BIPM, IEC, IFCC, ILAC, ISO, IUPAC, IUPAP and OIML, 120 pp, doi.org/10.59161/JCGM100-2008E.

3. Clegg, S. L.; Wexler, A. S., Densities and apparent molar volumes of atmospherically important electrolyte solutions. I. The solutes H_2SO_4, HNO_3, HCl, Na_2SO_4, $NaNO_3$, NaCl, $(NH_4)_2SO_4$, NH_4NO_3, and NH_4Cl from 0 to 50 °C, including extrapolations to very low temperature and to the pure liquid state, and $NaHSO_4$, NaOH and NH_3 at 25 °C. *J. Phys. Chem.* **2011,** *115*, 3393-3460.

4. Ford, T. D.; Call, T. G.; Origlia, M. L.; Stark, M. A.; Woolley, E. M., Apparent molar volumes and apparent molar heat capacities of aqueous 2-amino-2-hydroxymethyl-propan-1,3-diol (Tris or THAM) and THAM plus equimolal HCl. *J. Chem. Thermodyn.* **2000,** *32*, 499-516.

5. Tishchenko, P. Y., Non-ideal properties of the TRIS-TRIS.HCI-NaCI-H_2O buffer system in the 0-40 °C temperature interval. Application of the Pitzer equations. *Russ. Chem. Bull.* **2000,** *49* (4), 674-679.

6. Holmes, H. F.; Busey, R. H.; Simonson, J. M.; Mesmer, R. E.; Archer, D. G.; Wood, R. H., The enthalpy of dilution of HCl(aq) to 648 K and 40 MPa. Thermodynamic properties. *J. Chem. Thermo.* **1987,** *19*, 863-890.

7. Archer, D. G., Thermodynamic properties of the NaCl + H_2O system II. Thermodynamic properties of NaCl(aq), NaCl.2H_2O(cr), and phase equilibria. *J. Phys. Chem. Ref. Data* **1992,** *21*, 793-829.

8. Bates, R. G.; Macaskill, J. B., Activity and osmotic coefficients of t-butylammonium chloride: activity of HCl in mixtures with TRIS hydrochloride and t-butylammonium chloride at 25°C. *J. Solut. Chem.* **1985,** *14*, 723-734.

9. Harvie, C. E.; Weare, J. H., The prediction of mineral solubilities in natural waters: the Na-K-Mg-Ca-Cl-SO_4-H_2O systems from zero to high concentration at 25 C. *Geochim. et Cosmochim. Acta* **1980,** *44*, 981-997.

10. Millero, F. J.; Hershey, J. P.; Fernandez, M., The pK* of $TRISH^+$ in Na-K-Mg-Ca-Cl-SO_4 brines - pH scales. *Geochim. et Cosmochim. Acta* **1987,** *51*, 707-711.

11. Lodeiro, P.; Turner, D. R.; Achterberg, E. P.; Gregson, F. K. A.; Reid, J. P.; Clegg, S. L., Solid−liquid equilibria in aqueous solutions of Tris, Tris-NaCl, Tris-TrisHCl, and Tris-$(TrisH)_2SO_4$ at temperatures from 5 to 45 °C. *J. Chem. Eng. Data* **2021,** *66* (437-455).

12. Ji, X.; Lu, X.; Li, S.; L., Z.; Wang, Y.; Shi, J., Determination of the activity coefficients of NaCl in the system NaCl–NH_4Cl–H_2O. *J. Solut. Chem.* **2001,** *30* (5), 463-473.

13. Bates, R. G.; Bower, V. E., Standard potential of the silver-silver chloride electrode from 0 to 95 °C and the thermodynamic properties of dilute hydrochloric acid solutions. *J. Res. Natl. Bur. Stnds.* **1954,** *53*, 283-290.
